# Cell-cycle-independent transitions in temporal identity of mammalian neural progenitor cells

**DOI:** 10.1038/ncomms11349

**Published:** 2016-04-20

**Authors:** Mayumi Okamoto, Takaki Miyata, Daijiro Konno, Hiroki R. Ueda, Takeya Kasukawa, Mitsuhiro Hashimoto, Fumio Matsuzaki, Ayano Kawaguchi

**Affiliations:** 1 Department of Anatomy and Cell Biology, Nagoya University Graduate School of Medicine, 65 Tsurumai, Showa-ku, Nagoya, Aichi 466-8550, Japan; 2 Laboratory for Cell Asymmetry, Center for Developmental Biology, RIKEN Kobe Institute, 2-2-3 Minatojima-minamimachi, Chuo-ku, Kobe, Hyogo 650-0047, Japan; 3 Laboratory for Systems Biology, Center for Developmental Biology, RIKEN Kobe Institute, 2-2-3 Minatojima-minamimachi, Chuo-ku, Kobe, Hyogo 650-0047, Japan; 4 Functional Genomics Unit, Center for Developmental Biology, RIKEN Kobe Institute, 2-2-3 Minatojima-minamimachi, Chuo-ku, Kobe, Hyogo 650-0047, Japan; 5 Large Scale Data Managing Unit, Center for Life Science Technologies, RIKEN Yokohama Institute, 1-7-22 Suehiro-cho, Tsurumi-ku, Yokohama City, Kanagawa 230-0045, Japan; 6 Department of Anatomy, Fukushima Medical University, 1 Hikarigaoka, Fukushima 960-1295, Japan

## Abstract

During cerebral development, many types of neurons are sequentially generated by self-renewing progenitor cells called apical progenitors (APs). Temporal changes in AP identity are thought to be responsible for neuronal diversity; however, the mechanisms underlying such changes remain largely unknown. Here we perform single-cell transcriptome analysis of individual progenitors at different developmental stages, and identify a subset of genes whose expression changes over time but is independent of differentiation status. Surprisingly, the pattern of changes in the expression of such temporal-axis genes in APs is unaffected by cell-cycle arrest. Consistent with this, transient cell-cycle arrest of APs *in vivo* does not prevent descendant neurons from acquiring their correct laminar fates. Analysis of cultured APs reveals that transitions in AP gene expression are driven by both cell-intrinsic and -extrinsic mechanisms. These results suggest that the timing mechanisms controlling AP temporal identity function independently of cell-cycle progression and Notch activation mode.

## Abstract (web summary)

The molecular mechanisms determining the temporal identity patterns of self-renewing progenitors during cerebral development are largely unclear. Here, using single cell transcriptome analyses, the authors find progenitor temporal identity arises independent of cell-cycle progression and Notch activation.

## Graphical abstract

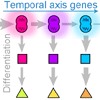

The functional organization of the brain requires the ordered generation of large numbers of diverse neurons and glia during development. The size and diversity of neural cell populations rely on the spatial and temporal diversity of progenitor cells. In mammalian cerebral cortex, self-renewing progenitor cells are formed by elongation of neuroepithelial cells, and repeated divisions at the apical surface of the ventricular zone (VZ) generate a stratified neuronal organization (these cells are thus termed apical progenitors (APs) or radial glial cells)^1^. Over time, these neural progenitor cells undergo temporal progression with respect to two properties (Fig. 1a). The first is the decision whether divisions are purely proliferative (expansive) or not. APs initially undergo proliferative divisions that generate two APs, and subsequently shift into a differentiating mode in which divisions give rise to non-AP cells, such as neurons^2^,^3^ or lineage-restricted intermediate progenitors (IPs)^1^,^4^. In the second, APs progressively change the fates of their differentiating progeny; deep-layer neurons→upper-layer neurons→glia^1^,^5^. The mechanisms underlying temporal patterns in neural progenitors are less well understood than those involved in the spatial patterning of these cells.

The transition of AP division mode from proliferative (symmetric) into differentiating (asymmetric) is not synchronized across the cerebral progenitor population. This shift initially takes place sporadically, and then progressively propagates into the entire brain with different timing. Cell-intrinsic programs and extrinsic environmental signals^6^,^7^ control these alterations in the division mode of APs^1^,^8^. Notch signalling is essential for progenitor self-renewal in both the proliferative and the neurogenic mode^9^,^10^. During the proliferative phase, the Notch ligand Delta-like 1 is mainly produced by APs, and is expressed in an oscillatory pattern^11^; subsequently, in the neurogenic phase, Delta-like 1 is produced by nascent IPs and neurons^12^,^13^. To date, however, it remains unclear how/when this temporal shift occurs in progenitor cells.

The molecular mechanisms underlying the temporal patterns of AP identity that generate sequential laminar fates of descendant neurons have been studied using a variety of approaches. *Foxg1* is involved in regulating the temporal progression of laminar fate potentials in a spatially controlled manner^14^. Genetic and epigenetic mechanisms are also involved in the transition from the neuronal to glial progenitors^15^,^16^,^17^. Transcriptome analyses have identified genes affecting temporal patterns in the AP identity^18^,^19^,^20^, providing lists of genes that exhibit dynamic temporal patterns in the VZ or neural stem cell population, but the fundamental features of the temporal progression of AP identity remain largely unknown. Is there an intrinsic timer mechanism that specifically counts and controls the progression of AP temporal patterns? Is such a mechanism coupled with cell-cycle progression or cytokinesis? How is the timing mechanism associated with environmental cues? An obvious difficulty in addressing these issues is that progenitors do not implement their temporal gene-expression patterns in a synchronized manner. In addition, gene-expression changes associated with cell-cycle progression overlie those that are exclusively involved in temporal progression of AP identity. Thus, the temporal progression of AP identity must be observed as a superposition of various time-dependent components. Transcriptome analysis at the single-cell level^13^,^21^,^22^,^23^ provides a unique opportunity to monitor variations in the gene-expression properties of progenitor cells. In combination with statistical analyses, this approach allows us to distinguish genes that are associated with temporal identity transition, the cell cycle, and/or differentiation, and thus provides clues regarding the relationships among those components.

In this study, we analyze comprehensive gene-expression profiles of single cortical progenitor cells at different developmental stages to explore the molecular bases of temporal patterns in AP properties, which generate shifts in division mode and differential laminar fate of progeny. In particular, we investigate changes in AP gene expression, as well as cell-to-cell variation in Notch signalling, over time. From the results of this analysis, we extract a set of genes whose expression levels are temporally altered, and then distinguish gene components that are purely associated with temporal progression in APs from those that are involved in the switch between self-renewal and differentiation. Moreover, we find that cell-cycle progression is not necessary for transitions in temporal gene expression and the laminar fate of APs. These findings, together with the results from *in vitro* culture of single APs, suggest the existence of a cell-autonomous ‘timer' in the APs, which is independent of the cell cycle and responsive to environmental cues.

## Results

### Gene-expression profiling identifies APs and IPs

**Figure 1:**
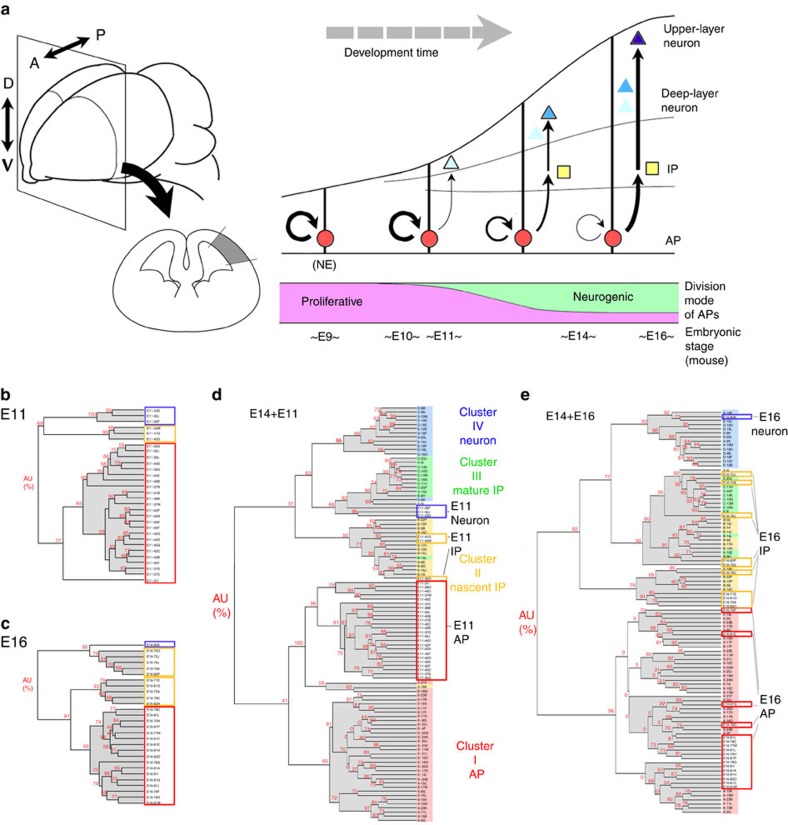
Classification of cortical progenitor cells. (**a**) Scheme of mammalian cerebral development. Before onset of neurogenesis, APs (apical progenitor cells, neuroepithelial cells (NEs) at this stage) undergo proliferative symmetric division. After onset of neurogenesis, APs overtime undergo temporal progression with respect to two properties: division mode (proliferative versus neurogenic) and the fates of their differentiating progeny (deep-layer neurons versus upper-layer neurons). A, anterior; P, posterior; D, dorsal; V, ventral; IP, intermediate progenitor cell. (**b**–**e**) E14-based hierarchical clustering analysis of single-cell cDNA classifies E11- and E16-derived cortical progenitor cells. Clustering dendrograms show the results from the SigABC genes. In the dendrograms, each label represents a single cell, and the label colour indicates the cluster where it belongs. The values in red at the branches are AU (approximately unbiased) *P* values (%). The horizontal branch length represents the degree of dissimilarity in gene expression among the samples. See also Supplementary Figs 1–4.

We compared global transcriptome profiles of single cells collected from murine dorsal cortices at three different developmental stages, E11 (proliferative or early neurogenic stage, 30 cells), E14 (mid neurogenic stage, 70 cells) and E16 (late neurogenic stage, 28 cells), by microarrays analysis of cDNAs from those single cells (Supplementary Fig. 1; Supplementary Table 1). In our earlier work^13^, we had analysed transcriptome profiles of the E14 cortical single cells, and successfully categorized the E14 cortical progenitor cells into two major populations, the APs and the IPs (or basal progenitors (BPs)), by hierarchical clustering using 114 probe sets; these probe sets, the ‘SigABC' genes, were identified by the first round clustering of the E14 single cells, supervised by a combination of known genes that distinguish self-renewal and commitment into IPs. We found that the same probe sets were applicable to classification of single E11 and E16 cells (Fig. 1b,c). We then determined how E11 progenitors were placed on the E14 progenitor cell cluster tree by combining the two populations of E11 and E14 single cells (Fig. 1d). The resultant dendrogram indicated that E11 progenitors could be categorized into either of the two major progenitor populations at E14. This also held true for the combination of ‘E14+E16' samples (Fig. 1e).

As a non-supervised analysis, we subsequently performed principal component analysis (PCA) on progenitor samples at each stage, using all available probe set data (17,192 probe sets, Supplementary Fig. 2). The principal component 1 (PC1) axis, along which the variance in gene expression is greatest among the tested cells, clearly reflected the differentiation state of progenitors, APs and IPs, further confirming the validity of our classification of the progenitor cells.

Because the Notch signalling pathway is the canonical signalling pathway that is differentially expressed between the IPs and APs from E11 to E14 (ref. 13) and E16 (Supplementary Fig. 3), we investigated the temporal change in the Notch signalling status of the progenitors by performing PCA focused on 21 genes related to Notch signalling (Supplementary Fig. 4). Interestingly, the expression of these Notch signalling genes shifted from highly variable to relatively uniform among individual progenitors as development proceeded from E11 to E16. This change in Notch signalling is consistent with the shift in Notch activation mode from mutual activation among APs at the proliferative state^9^,^11^ into the lateral inhibition mode in the neurogenic state^12^,^13^ (see Discussion).

### Developmental pattern of gene-expression profiles of APs

**Figure 2:**
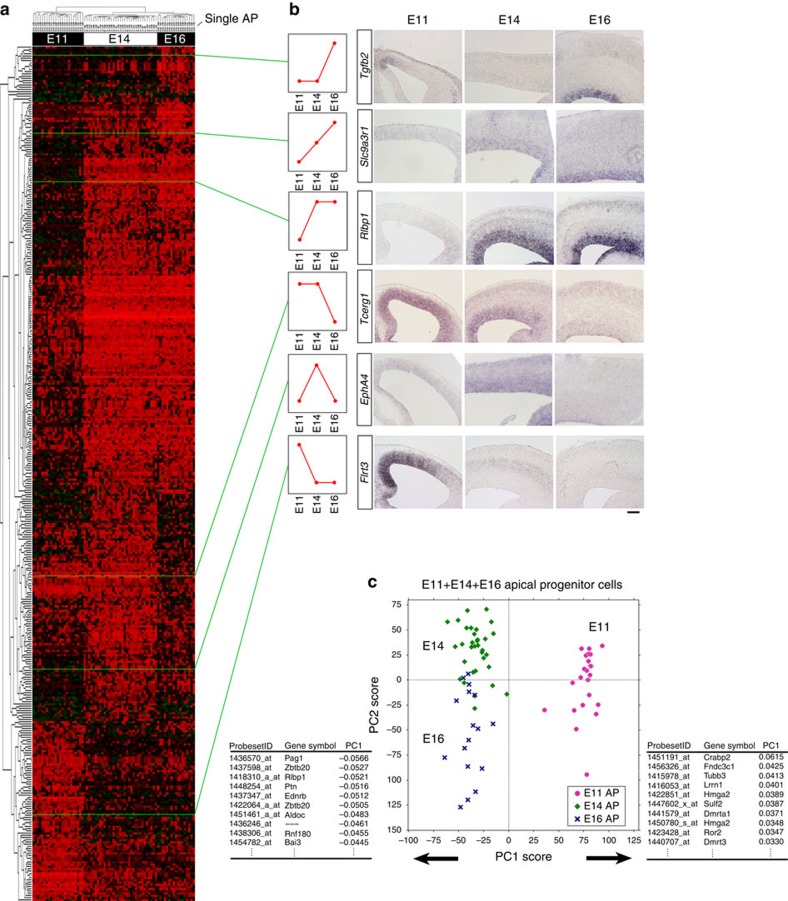
Temporal change in gene expression in APs. (**a**) Two-way cluster analysis of genes differentially expressed among E11, E14 and E16 single-cell cDNAs of the APs (384 probe sets, Supplementary Data 1, selected by one-way ANOVA). Examples of genes that exhibited typical expression patterns in E11, E14 and E16 cerebrum are shown in **b** or Supplementary Figs 5 and 6. The ‘medial>lateral' genes exhibited an ‘E11>E14' trend, and the ‘medial<lateral' genes exhibited an ‘E11<E14' trend (Supplementary Fig. 7). Scale Bar, 100 μm (**c**) Global gene-expression patterns of E11 APs are very different from those of E14/E16 APs. PCA was performed on microarray data from single-cell cDNAs of all APs (total *N*=73: mixture of E11, *N*=23; E14, *N*=33; E16, *N*=17 single cells; 17192 probe sets). Each symbol indicates one cell. PC1, the most representative axis for the gene-expression variation among the AP population, is determined by the difference between E11 and E14/E16 cells. The lists below indicate the top 10 genes that positively or negatively influence PC1. Proportion of variance: 0.0436 (PC1) and 0.0319 (PC2). See also Supplementary Figs 5–9 and Supplementary Data 1.

Because the temporal pattern of gene expression in self-renewing progenitors could play important roles in transitions of cortical progenitor states as well as the laminar fates of descendant neurons^1^, we examined temporal changes in AP gene expression. To this end, we first selected the genes that showed significantly differential expression among E11, E14 or E16 single APs (Supplementary Data 1) (384 probe sets, selected by analysis of variance (ANOVA), false discovery rate (FDR)<0.1) (genes that showed significantly differential expression among the E11, E14, or E16 single IPs are presented in Supplementary Data 2). As shown in Fig. 2a, the majority of these genes changed their expression levels between E11 and E14 (303 probe sets among the 384 selected probe sets), as exemplified by *Flrt3* (E11>E14) and *Rlbp1* (E11<E14) (Fig. 2b; Supplementary Fig. 5). A smaller number of genes changed later between E14 and E16 (107 probe sets among the 384 selected probe sets), as *Tcerg1* (E14>E16) and *Tgfb2* (E14<E16) did (Fig. 2b; Supplementary Fig. 6). Among these genes, several genes were expressed in a mediolateral gradient (Supplementary Fig. 7).

We performed PCA using all APs of the three stages (total *N*=73 cells) to objectively evaluate the temporal changes in global gene expression in APs (Fig. 2c; Supplementary Fig. 8). In the scatter diagram of PC1/PC2 scores, PC1 clearly revealed a large difference between the E11 and E14 cell populations, consistent with the comparison described above. The top 10 genes that contributed positively or negatively to PC1 (Fig. 2c) included those involved in the control of heterochronic genes, such as *Hmga2* (ref. 24), as well as ‘temporal-axis' genes (see later).

Our results indicate that APs undergo large genome-wide changes in gene expression between E11 and E14, rather than between E14 and E16 (see later sections). To evaluate how APs change their characteristics between E11 and E14, we performed GO analysis of the top 150 probe sets that contributed positively or negatively to PC1. According to functional annotations^25^, many E11>E14/E16 genes (‘PC1-positive' genes) were related to ‘nucleoprotein biogenesis', ‘cancer pathway' or ‘cell cycle' (Supplementary Table 2). Therefore, the genome-wide transition in the AP state from E11 to E14 appears to involve a shift in the proliferation mode of APs. On the other hand, many E11<E14/E16 genes (‘PC1-negative' genes) are related to ‘polysaccharide binding', ‘extracellular matrix', or ‘cell adhesion', suggesting that between E11 and E14, progressive changes occur in the extracellular environments surrounding APs.

### Identification of ‘temporal axis' and ‘differentiation axis'

**Figure 3:**
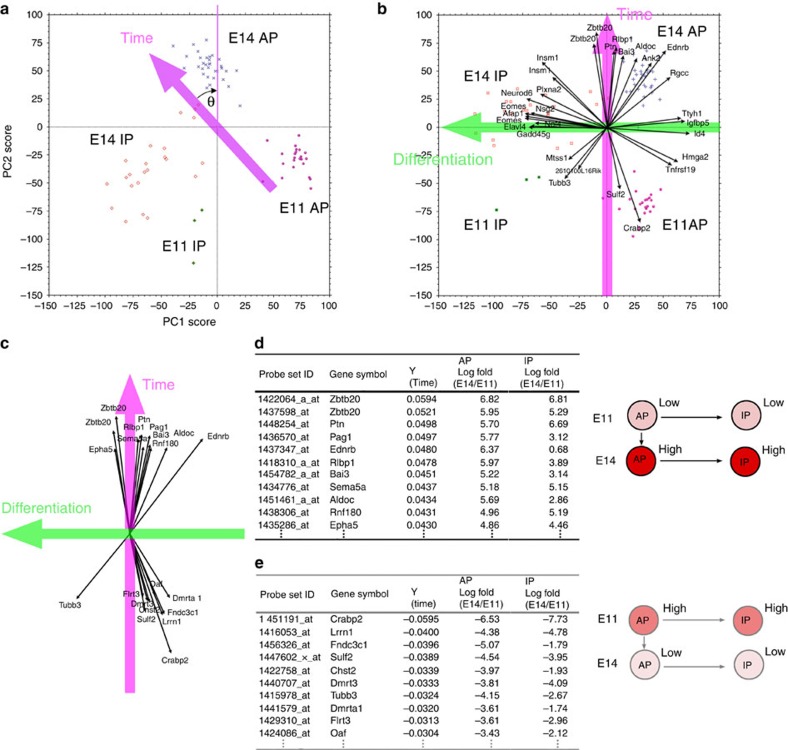
Expression change in an individual gene can be described by the temporal and differentiation axes. (**a**) PCA was performed using microarray data from single-cell cDNAs of E11 and E14 progenitor (AP+IP) cells (E11, *N*=23; E14, *N*=56; 17,192 probe sets). Each symbol represents one cell. Plots for individual cells, categorized into one of four groups (E11 APs, E11 IPs, E14 APs and E14 IPs), are almost completely separated in a two-dimensional PCA graph. To make the new *Y* axis the temporal axis, graph **a** was rotated such that the median new *X* values of E11 APs and E14 APs were equal (θ≈43.35°) (graph **b**). (**b**) Distribution of four progenitor groups and vectors of the 30 genes with the largest vectors showed that the new *X* axis primarily reflects differences between APs and IPs, and thus represents the differentiation axis. (**c**–**e**) Temporal-axis genes. The top 10 genes that made the largest positive or negative contributions to the temporal axis were selected and shown as a vector (**c**) or in tables (**d**,**e**). These genes roughly overlapped with the main PC1 genes from PCA of E11, E14, and E16 APs (Fig. 2c). Log fold-change values of expression levels showed that these genes exhibited an E11-low/E14-high pattern (or vice versa) in both APs and IPs.

As we described above, Notch signalling status changes over time in cortical progenitors; hence, the temporal changes in AP gene expression must include the consequences of a shift in the differentiation status of APs. Therefore, we tried to distinguish exclusively temporal components from those related with the differentiation status of APs. As the first step in doing so, we incorporated information related to the differentiation status of all progenitors into our global transcriptional profiling of progenitors by performing PCA for all progenitor populations (AP and IPs) at both E11 and E14 (*N*=82). Within the coordinates of PC1 and PC2 (Fig. 3a; Supplementary Fig. 9), which represent two major types of variation among samples, individual cells were distributed as four separate groups (E11 APs, E11 IPs, E14 APs and E14 IPs).

We found that a rotation of the coordinates in the PC1–PC2 diagram clarified the temporal axis and differentiation axis (Fig. 3b). In particular, the coordinates were rotated such that the temporal axis of the AP population became parallel with the vertical axis, i.e., so that the median *X* value of the E14 APs was equal to that of the E11 APs. Such a rotation of the PC1–PC2 coordinates also rotates the vectors that represent the contribution of each gene to the PC1–PC2 coordinates (Fig. 3b). In the new coordinate system following the rotation, genes that contributed highly to the horizontal axis (or nearly parallel to the horizontal axis) turned out to include those reflecting differences between APs and IPs^13^, such as *Eomes* (*Tbr2*), *Gadd45g* and *Ttyh1* (Supplementary Fig. 3). Concomitantly, IPs and APs were distributed along this new horizontal axis. Thus, we conclude that the rotation of the axes such that the vertical axis is interpreted as the temporal axis causes the horizontal axis to represent the differentiation status of progenitor cells. Of note, these two axes are orthogonal to each other, implying the underlying biological phenomena are separable.

Based on these statistical procedures, we were able to identify genes that contributed highly to the temporal axis, either negatively or positively, as the ‘temporal-axis genes'. Lists of the top 10 genes are shown in Fig. 3c–e. Interestingly, changes of the temporal-axis genes in expression from E11 to E14 tend to be in parallel between APs and IPs; genes expressed at low levels in E11 APs but high levels in E14 APs also exhibited a similar pattern in IPs (low at E11; high at E14), and vice versa, as shown in Fig. 3d,e. This observation suggests that temporal information, represented by these temporal-axis genes, is inherited by IPs from APs.

### APs change their gene-expression pattern mainly in E12

**Figure 4:**
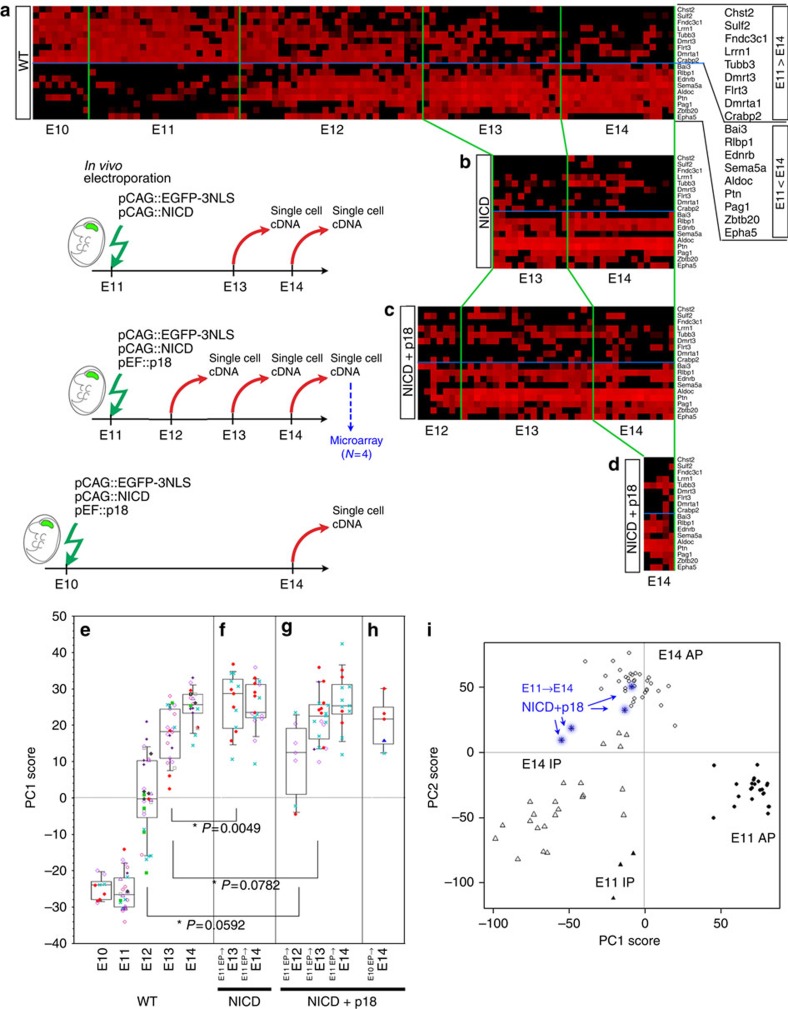
Neither Notch activation nor cell-cycle arrest stops the temporal change in gene expression of APs. (**a**–**d**) Expression levels of 18 temporal-axis genes in single APs examined by qPCR. [40-Ct] values after normalization by *Gapdh* range from high (red) to low (undetectable) (black) in these heat maps. One column indicates a single AP. (**a**) Single-cell cDNAs from E10–14 wild-type APs. (**b**–**d**) NICD (**b**), or NICD and p18 (**c**) were overexpressed along with EGFP at E11; the genes were introduced by *in vivo* electroporation. After 1 day (E12), 2 days (E13) or 3 days (E14), single-cell cDNAs were generated from EGFP^+^ cells. APs were selected as *Ttyh1*^*+*^*/Tbr2*^*−*^. (**d**) NICD and p18 were overexpressed along with EGFP at E10; the genes were introduced by *in vivo* electroporation. After 4 days (E14), single-cell cDNAs were generated from EGFP^+^ cells. APs were selected as *Ttyh1*^*+*^*/Tbr2*^*−*^. (**e**) PC1 scores of single APs calculated from the expression levels of 18 temporal-axis genes. PCA was performed on normalized [40-Ct] values of 18 genes in a mixture of E10–E14 APs (*N*=102), and the PC1 scores of single APs were plotted at each developmental stage. One dot indicates one AP, and cells from the same embryos are indicated by the use of the same symbols in each stage. See also Supplementary Fig. 10. (**f**–**h**) PC1 scores of NICD-expressing (**f**), or NICD/p18 co-expressing APs (**g**,**h**) were calculated using Component 1 obtained from PCA on wild-type APs (**e**). (**i**) PCA was performed using the microarray data from single-cell cDNAs from NICD/p18-overexpressing cells (E11 electroporation, E14 sampling, *N*=4, blue) and E11 and E14 progenitor cells (APs+IPs, *N*=79; 17,192 probe sets). Each symbol represents one cell. The two plots for the NICD/p18 cells are located among the plots for the E14 APs. The other two plots for the NICD/p18 cells are located near the plot for the E14 IPs, which may reflect the effect of p18 on differentiation (Fig. 5b). See also Supplementary Fig. 13.

Using these temporal-axis gene sets, we analysed detailed temporal patterns in the gene expression of APs from E11 to E14. To do so, we generated additional single-cell cDNAs from E10, E12, and E13 cerebral walls, and selected single APs as *Ki67*^+^/*Ttyh*1^+^/*Tbr2 (Eomes)*^−^ cells by quantitative real-time PCR (qPCR), as this combination of markers reliably identifies APs^13^ (Fig. 3b). We then examined the expression levels of 18 temporal-axis genes (Fig. 3c–e) in those single-cell cDNAs. As shown in Fig. 4a, these genes changed their expression levels in the AP population gradually during development. This gradual change appears to proceed in individual APs, because APs at E12 and E13 frequently co-expressed both E11-type genes (including transcription factors such as *Dmrt3* and *Dmrta1*) and E14-type genes (such as the *Zbtb20* transcription factor). We then statistically evaluated how the 18 temporal-axis genes changed their expression levels in APs over time, by performing PCA for these genes for all APs (mixture of E10–E14, *N*=102) (Fig. 4e; Supplementary Fig. 10). The PC1 scores of APs showed the widest distribution at E12 during E10 and E14, indicating that the temporal-axis genes mainly changed their expression around E12. Taken together, these results suggest that the transition in temporal-axis gene expression gradually proceeds in individual APs around E12. At around this stage, the shift from the proliferative to neurogenic character occurs gradually^26^. This, and the results of the GO analysis of PCA for all APs, suggest that the major shift in the temporal-axis genes associates with the transition from the proliferative state to the neurogenic state (see Discussion).

### Cell-cycle arrest does not prevent AP transitions

**Figure 5:**
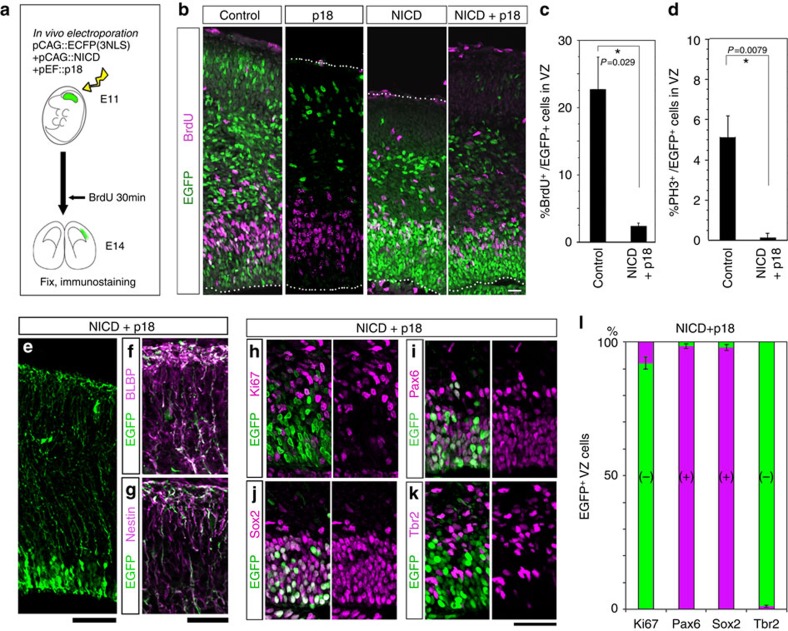
Co-electroporation of Cdk inhibitor p18 and NICD inhibit cell-cycle progression of progenitors while maintaining them in an undifferentiated state. (**a**) Experimental design. pCAG::EGFP-3NLS alone (control), or with pCAG::NICD, or with both pCAG::NICD and pEF::p18 was electroporated into E11 cerebral wall *in vivo*. At E14, BrdU was administered for 30 min, and brains were fixed and stained with antibodies to EGFP, BrdU (**b**,**c**), PH3 (**d**), BLBP (**f**), Nestin (**g**), Ki67 (**h**), Pax6 (**i**), Sox2 (**j**) or Tbr2 (**k**). (**c**,**d**) Frequency of BrdU^+^ cells (**c**) or PH3^+^ cells (**d**) in the EGFP^+^VZ cells was significantly reduced relative to the control by NICD/p18 co-overexpression (**c**, *N*=4 for each case; **d**, *N*=5 for each case; Mann–Whitney *U* test, means±s.d.). The characterization of the EGFP^+^ cells in the IZ/SVZ and CP is presented in Supplementary Fig. 11. (**e**–**g**) NICD/p18 co-expressing cells have radially elongated fibres that are positive for BLBP and Nestin. pCAG::EGFP, pCAG::NICD and pEF::p18 were co-electroporated on E11, and the brains were examined at E14. (**h**–**l**) Most of the NICD/p18 co-expressing cells in the VZ are negative for Ki67 and Tbr2 and positive for Pax6 and Sox2. Bars, 20 μm in (**b**), 60 μm in (**e**), 30 μm in (**f**,**g**), 40 μm in (**h**–**k**).

We next investigated whether cell-cycle progression was necessary for temporal changes in AP gene expression. Overexpression of cyclin-dependent kinase (Cdk) inhibitor arrests the cell cycle of cortical progenitor cells, and also leads to precocious differentiation^27^. Therefore, the simultaneous overexpression of the Cdk inhibitor Cdkn2c (p18)^28^ and the intracellular domain of Notch1 (NICD) should arrest the cell cycle of APs, because *NICD* maintains self-renewal potential^29^,^30^. When the E11 or E12 cerebral wall was electroporated with *NICD* and *p18*, together with *Egfp* (Fig. 5a), the majority of EGFP^+^ cells were confined to the E14 VZ (Fig. 5b; Supplementary Fig. 11), consistently with the radially elongated Nestin^+^ and BLBP^+^ fibres (Fig. 5e–g). These EGFP^+^ cells were positive for the AP markers Pax6 and Sox2 (refs 31, 32, 33), (Fig. 5i,j,l) and negative for Tbr2 (Fig. 5k,l), a marker of differentiating cells^33^. On the other hand, the proportions of BrdU^+^ S-phase cells and PH3^+^ M-phase cells were significantly reduced in EGFP^+^ cells relative to control cells (Mann–Whitney *U* test, Fig. 5c,d). In addition, Ki67 immunoreactivity was rarely seen (Fig. 5h,l). These results demonstrated that NICD/p18 co-overexpression successfully blocked cell-cycle progression of APs while maintaining AP properties.

We then investigated the effects of simultaneous expression of NICD and p18 on temporal gene expression of APs by focusing on the 18 temporal-axis genes described above (Fig. 3c–e). We electroporated the cerebral wall at E10 or E11 with *NICD* with or without *p18* (and *Egfp*, to allow visualization of the electroporated cells), and then generated cDNAs from single EGFP^+^ cells at E12, E13 or E14 (Fig. 4b–d). We identified APs retrospectively, by qPCR, as *Egfp*^*+*^*/Ttyh1*^*+*^*/Tbr2*^−^ cells, and confirmed the absence of *Ki67* expression in these cells after co-electroporation of *NICD* and *p18*, indicative of cell-cycle arrest. Thereafter, we examined expression levels of 18 temporal-axis genes by qPCR (Fig. 4b–d), and calculated the PC1 score for each AP cell (Fig. 4f–h) to depict overall changes from E12 to E14 in the gene expression of the AP population using PCA Component 1, which was obtained from wild-type APs at E10–E14 (Fig. 4e).

PC1 scores of NICD-expressing APs at E13 were higher than those of wild-type E13 APs (Fig. 4e,f, *P*=0.0049), suggesting that constitutive activation of Notch signalling affected expression levels of these temporal-axis genes to some degree (for example, decreased *Sema5a* expression). Nonetheless, NICD/p18 co-expressing APs clearly exhibited a temporal transition of PC1 scores from E12 to E14 (Fig. 4g,h), indicating that, even when cell-cycle progression was arrested, APs underwent temporal changes in gene expression. At E13 or E14, PC1 scores in NICD/p18 co-expressing APs were not distinguishable from those in APs solely expressing NICD (Fig. 4f,g, *P*=0.119 at E13; *P*=0.615 at E14). Similar results were also obtained after NICD/Cdkn1b (p27) co-expression (Supplementary Fig. 12). Therefore, cell-cycle arrest does not appear to have an additional effect beyond that of NICD on the expression of temporal-axis genes.

We also examined the gene expression profiles of NICD/p18 co-expressing APs by using microarray (*N*=4). PCA on these cells with E11+E14 progenitors indicates that their temporal progression is not arrested (Fig. 4i; Supplementary Fig. 13). Together, these results strongly suggest that cell-cycle progression is not necessary for the transition of temporal identity genes in APs between E10/11 and E14.

### Effect of transient cell-cycle arrest on laminar fate

Our results regarding the effect of the cell cycle on temporal-axis genes are not consistent with previous studies that argued that the laminar fate of neurons is correlated with the number of cell cycles that their progenitors have executed *in vivo*^34^. Therefore, we investigated whether cell-cycle arrest would affect the temporal laminar fate transition in APs in our experimental system.

**Figure 6:**
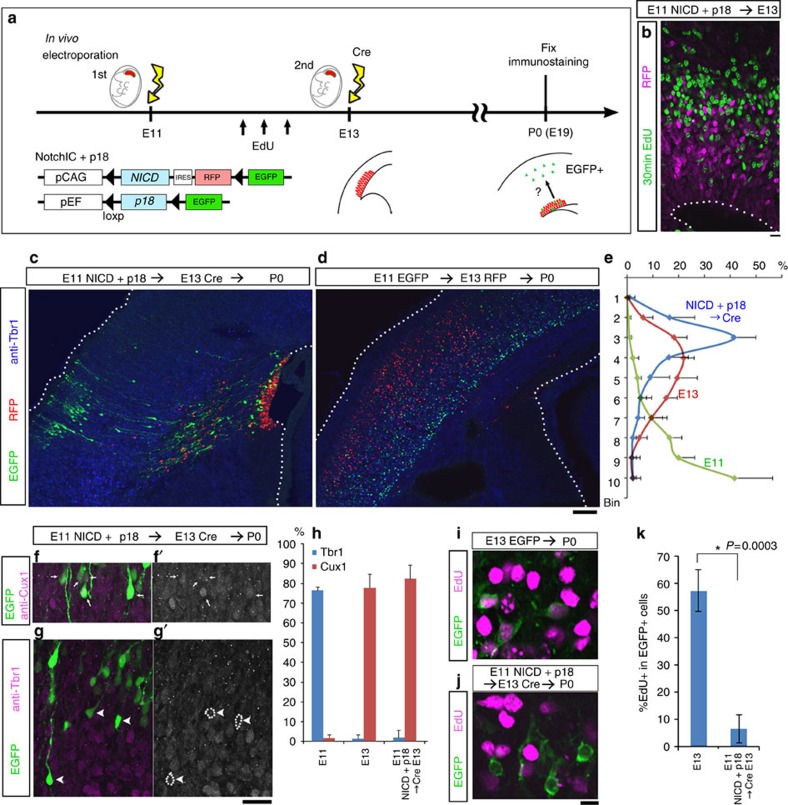
Laminar fate of progenitor cells is not altered by transient cell-cycle arrest. (**a**) Experimental design of double *in vivo* electroporation study. (**c**) RFP^+^ cells did not label with EdU administered 30 min before sacrifice at E13. Bar, 10 μm. (**c**,**d**) Immunostaining with antibody to Tbr1 (blue) of sections from E11 NICD+p18/E13 Cre double-electroporated P0 brain (**c**) or control E11 EGFP-3NLS/ E13 RFP-3NLS double-electroporated P0 brain (**d**). Scale bar, 100 μm. (**e**) Distribution of the electroporated cells in the CP (cortical plate). CP was separated into 10 bins, and Bin1 represents the most exterior portion of the CP. Data show means±s.d. from *N*=6 embryos for each experimental condition. (**f**,**g**) Cux1 (**f**, **f**′) or Tbr1 (**g**, **g**′) immunoreactivity in E11 NICD/p18 and E13 Cre double-electroporated P0 brain. Arrows (**f**, **f**′) indicate EGFP^+^/Cux1^+^ cells, and arrowheads (**g**, **g**′) indicate EGFP^+^/Tbr1^−^ cells in the CP. (**h**) Frequency of Cux1+ or Tbr1^+^ cells in electroporated cells in the CP. Data show means±s.d. from *N*=4, 5, 4 (Cux1 data) and 4, 4, 6 (Tbr1 data) embryos for E11, E13, NICD/p18/Cre, respectively. (**i**–**k**) EdU was administrated three times at 4-hr intervals at E12, and labelling index was examined at P0 CP. Many EGFP^+^ CP cells that had been electroporated with pCAG::EGFP-3NLS at E13 incorporated EdU (**i**), whereas E11 NICD/p18 and E13 Cre double-electroporated CP cells exhibited no significant incorporation (**j**). Data show means±s.d. *P=0.0003, *N*=7 and 8 embryos, Mann–Whitney *U* test. See also Supplementary Fig. 14.

Cortical progenitors overexpressing NICD at the early neurogenic stage fail to produce early born neurons, but start to generate late-born neurons instead of early born neurons if NICD overexpression is halted 2 days later^29^. Modifying this strategy, we examined the laminar specificity adopted by neurons after cortical progenitors experienced temporal cell-cycle arrest due to the activation of Notch and p18 (Fig. 6), because NICD alone did not arrest cell-cycle progression of the progenitor cells (Fig. 5b). If early born neurons were generated from cortical progenitors after temporary Notch/p18 activation, then it would imply that cell-cycle progression is required in order for cortical progenitors to proceed with the transition of laminar fate. On the contrary, if late-born neurons were generated after the same treatment, it would indicate that cell-cycle arrest does not affect progressive changes in the laminar fate of neurons. For temporary expression of both NICD and p18 in APs, the activation and termination of the two genes were controlled by a double *in vivo* electroporation method^29^ based on the Cre-recombinase-loxP system (Fig. 6a): the first electroporation for expression plasmids of NICD and p18, and the second one for Cre-recombinase expression. At E11, we performed the initial *in vivo* electroporation to express p18 and NICD, along with RFP. After 2 days, the second electroporation was performed at E13 to express Cre recombinase, which excised *p18*, *NICD* and *RFP* cDNA sequences at the flanking loxP sites to allow EGFP expression in double-electroporated cells (Fig. 6a). At E13, we confirmed, by 30-min EdU labelling, that RFP^+^ cells were not cell cycling (Fig. 6b). Six days after the second electroporation (at E19/P0), most RFP^+^ cells (NICD/p18 co-expressing APs under cell-cycle arrest) were still confined to the VZ, whereas EGFP^+^ double-electroporated cells were present in the cortical plate (CP) and intermediate zone (Fig. 6c). These EGFP^+^ cells in the CP were positioned in the upper layers (Fig. 6e), and they were positive for the upper-layer marker Cux1 (ref. 35) and negative for the deep-layer marker Tbr1 (ref. 36) (Fig. 6f–h), suggesting that these cells were upper-layer neurons. The positions of EGFP^+^ cells were farther outside the CP than control RFP^+^ cells that had been electroporated at E13 (Fig. 6c–e); a similar result was obtained when the initial electroporation was performed at E10 (Supplementary Fig. 14). We reasoned that double-electroporated cells took time to resume cell-cycle progression and differentiation. Consistent with this, 15.1±1.9% of EGFP^+^ CP cells were EdU^+^ when pulse labelled at E14, one day after the second electroporation (Supplementary Fig. 14). We also confirmed that the EGFP^+^ CP cells rarely incorporated EdU when administered three times at 4 h intervals at E12 (Fig. 6a,i–k), indicating that these cells had undergone arrested cell-cycle progression at E12.

Together, these results suggest that transient cell-cycle arrest *in vivo* does not interfere with the laminar fate transition of APs from deep layers to upper layers during the neurogenic phase.

### Transition of temporal-axis genes in clonally maintained APs

**Figure 7:**
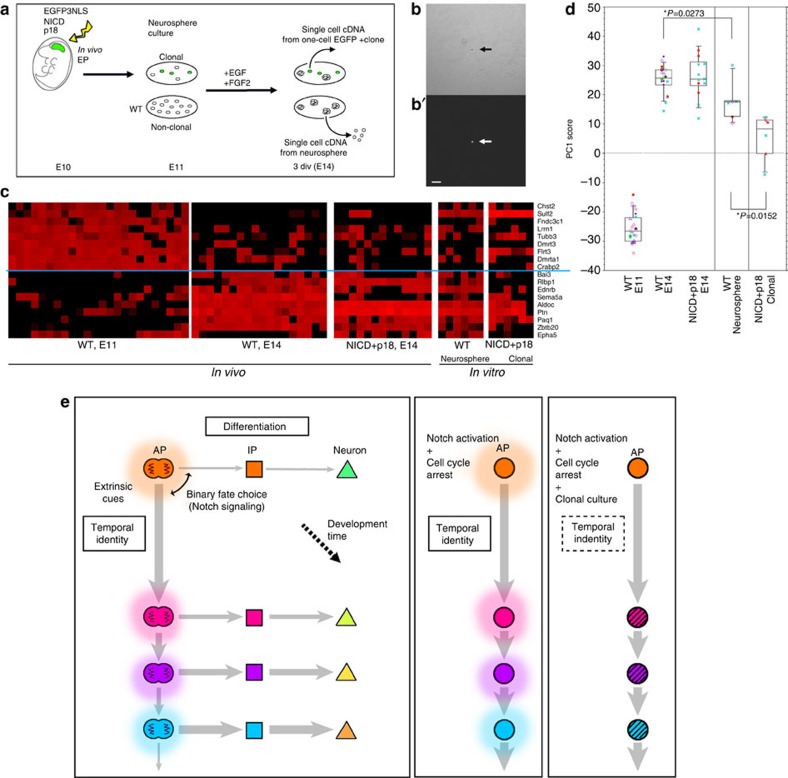
Temporal change of gene expression in APs is partly cell autonomous. (**a**) Experimental design. (**b**) Single EGFP^+^ cell (arrow) formed one-cell clone at 3 *div* in clonal culture. b, Bright field; b′, EGFP fluorescence. Scale bar, 50 μm. (**c**) Expression levels of 18 temporal-axis genes in single APs examined by qPCR. Levels range from high (red) to low/undetectable (black) in these heat maps. Each column indicates a single AP (*N*=24, wild-type E11 APs; *N*=18, wild-type E14 APs; *N*=13, E14 APs electroporated with NICD/p18 at E11 (identical to the data shown in Fig. 4); *N*=6, APs from neurospheres; *N*=6, NICD+p18 co-expressing APs from one-cell clones. NICD+p18 co-expressing APs from one-cell clones were *Egfp*^*+*^*/Ttyh1*^*+*^*/Sox2*^*+*^*/Hes5*^*+*^*/Tbr2*^*−*^*/Ki67*^*−*^, as determined by qPCR. (**d**) PC1 scores of wild-type APs at E11 and E14, NICD/p18 co-expressing APs at E14 (identical to the data shown in Fig. 4), APs from neurospheres, and NICD+p18 co-expressing APs from one-cell clones, which were calculated using Component 1 obtained from PCA of wild-type APs (Fig. 4e). Note that the PC1 scores for the neurosphere-derived APs differ significantly from those for the wild-type E14 APs (*P*=0.0273, Mann–Whitney *U* test). See also Supplementary Fig. 16, which shows the characterization of the microarray data from the single neurosphere-derived APs. (**e**) Temporal patterns of cortical progenitor cells (model). Transition of temporal identity of APs, which occurs gradually over the course of development, cannot be stopped by constitutive Notch activation or cell-cycle arrest *in vivo*. This transition of temporal identity includes both the transition in division patterns and transition in laminar fate potential of APs. Clonal culture of cell-cycle-arrested APs partly impairs transition of temporal gene expression, suggesting that the transition in temporal identity is regulated by both cell-autonomous and non-cell-autonomous mechanisms.

Finally, we investigated to what extent the temporal change in AP gene expression was controlled in a cell-autonomous manner. For this purpose, ideally no cell should be in contact with individual APs. Thus far, this type of study has not been successfully performed, because APs proliferate to generate clones of two or more cells, and non-cell-autonomous or contact-dependent effects from sibling cells could not be excluded even in clonal culture. By contrast, NICD/p18 co-expressing APs can maintain the one-cell state *in vitro* (Fig. 7a,b), thus enabling for the first time a stringent examination of cell-autonomous effects.

We introduced *NICD* and *p18* (together with *Egfp*) into the cerebral wall of E10 mice (Fig. 7a); 1 day later, the cells were dissociated and cultured at clonal density. Three days after *in vitro* culture, we obtained cDNAs from each of the single EGFP^+^ cells from one-cell clones (Fig. 7b), and identified APs as *Ttyh1*^+^/*Tbr2*^−^ cells to examine the expression levels of 18 temporal-axis genes by qPCR. To evaluate the effects of culture media on temporal gene expression, we also isolated single AP cells from neurospheres grown in the same media.

In APs from neurospheres (*Ki67*^*+*^*/Sox2*^*+*^*/Pax6*^*+*^*/Ttyh1*^*+*^*/Tbr2*^−^ cells), the expression levels of some temporal-axis genes were similar to those in E14 APs *in vivo*, whereas the expression levels of other genes were not (Fig. 7c,d). For example, *Sulf2* was expressed both in all neurosphere-derived APs and in all NICD/p18 co-expressing clonal APs, more frequently than in *in vivo* APs (*P*=0.0006 and *P*=0.0031, respectively, Fisher's exact test), suggesting that *Sulf2* expression was affected by the culture media. The expression levels of 18 temporal-axis genes differed significantly between the neurosphere-derived APs and APs *in vivo* (PC1 scores, *P*=0.0273, Mann–Whitney *U* test). Together, the expression of the temporal-axis genes and the results of the microarray global gene expression analysis (Supplementary Fig. 15) suggest that the neurosphere-derived APs partially but not precisely reflect the *in vivo* changes in the temporal gene-expression levels of APs.

However, the changes in gene expression in NICD/p18 co-expressing AP clones were more limited than those observed in neurosphere-derived APs (Fig. 7d, *P*=0.0152, Mann–Whitney *U* test). For example, *Zbtb20*- or *Rlbp1*-expressing cells, which emerged *in vivo* as well as in neurosphere-derived APs, were not observed in NICD/p18 co-expressing clonal APs (*P*=0.039 for *Zbtb20*, *P*<0.0001 for *Rlbp*1, NICD/p18 clonal APs versus NICD/p18 E14 *in vivo* APs, Fisher's exact test). These results suggested that non-cell-autonomous mechanisms regulated the expression of these temporal-axis genes. Surprisingly, even in NICD/p18 co-expressing clonal APs, several genes exhibited temporal expression patterns similar to those seen *in vivo*, including a decrease of *Dmrta1, Dmrt3, Crabp2, Lrrn1* and *Fndc3c1*, and an increase of *Aldoc, Ptn* and *Pag1*, from E11 to E14. Since we selected clonal NICD/p18 expressing cells that were isolated from other cells by >400 μm, these results suggest that temporal changes in the expression of these genes are controlled in a cell-intrinsic manner.

We also examined the laminar fate potentials of two different types of cultured APs *in vitro*; one-cell AP clones co-expressing NICD/P18 (Supplementary Fig. 16) and APs from neurospheres. The one-cell AP clones were prepared by *in vitro* culture after Cre-dependent NICD/P18 expression plasmids were introduced into the E11 cortex as previously described (Fig. 6), and this was followed by the excision of NICD/P18 via virus-mediated introduction of Cre-recombinase (Fig. 6a) after 2 days of low-density culture of E12 cells (Supplementary Fig. 16). We first examined the laminar cell fates of the neurons in the neurospheres derived from the E11 APs (formed in the absence of NICD/P18) as an *in vitro* culture control (Supplementary Fig. 16). When the neurons from the neurospheres were examined in 8 *div* differentiated monolayer cultures produced from 3 *div* neurosphere cultures, 19% of the cells were differentiated into neurons (TuJ1+), and 20% of the neurons expressed Cux1. In contrast, when the one-cell AP clones that had been temporally arrested by NICD/P18 expression were allowed to restart neurogenesis via excision of NICD/P18, 6% of the neurons were Cux1^+^, whereas Tbr1 was expressed in 16% of the neurons. Thus, the subset of the one-cell AP clones in which the cell cycle was transiently arrested (for approximately E11–13) *in vitro* appeared to generate neurons with upper-layer identity when neurogenesis restarted, although the rate of upper-layer neuron production was low compared with that in the *in vivo* experiments (Fig. 6). Thus, the differentiation assays of the one-cell AP clones cultured *in vitro* (Supplementary Fig. 16) suggest that transitions of laminar fate potential occur at low frequency and incompletely in the *in vitro* single-cell state, even when the cell cycle is temporally arrested. This conclusion is consistent with the expression of temporal-axis genes (Fig. 7).

Taken together, our observations suggest that the cell-cycle-independent transitions of the AP temporal genes are controlled by both cell-autonomous and non-cell-autonomous mechanisms.

## Discussion

Our genome-wide transcriptome profiling of single cells revealed temporal changes that occur in APs and IPs from E10 to E16. Using statistical methods, we resolved these changes into two components, which we refer to as the temporal and differentiation axes. These two axes are orthogonal (Fig. 3), and changes along the differentiation axis are correlated with Notch signalling. Thus, temporal changes in the expression of genes are, in principle, separable from the Notch signalling pathway.

During the period examined in this study, the APs gradually underwent changes in global gene expression (Fig. 2; Supplementary Fig. 8), which primarily occurred at approximately E12, as revealed by the shift in the expression of the temporal-axis genes (Fig. 4). This shift is most probably associated with the transition from the proliferative state to the neurogenic state for the following three reasons. First, a recent study using the mosaic analysis with double markers system has suggested that the switch from proliferative to neurogenic division occurs once in each progenitor cell lineage and has also indicated that neurogenic AP divisions are minor (31%) at E11, but become major (74%) at E12 (ref. 26). These results indicate that the proliferative to neurogenic transition progresses at approximately E12. Second, from this stage onward, the rate of mitosis in the subventricular zone (SVZ), perhaps reflecting that of IPs, increases^3^, suggesting that the change in cell fate from proliferative APs to neurogenic IPs begins at this stage. These observations coincide closely with the timing of progressive changes in temporal-axis gene expression. Third, many of the temporal-axis genes that undergo a transition at E12 are involved in cell proliferation. For example, the E11-type temporal-axis gene *Sulf2*, which encodes a sulfatase that edits the sulfation status of heparan sulfate proteoglycans, is broadly upregulated in many cancers^37^. Embryos in which the E14-type temporal-axis gene, *Ptn* (*pleiotrophin/HB-GAM*), is knocked out exhibit an elevated rate of proliferation during cortical neurogenesis, whereas exogenous Ptn inhibits the formation and growth of FGF-2-stimulated neurospheres^38^. Another E14-type temporal-axis gene, *Pag1 (Cbp*), encodes a transmembrane adaptor protein that functions as a suppressor of Src-mediated tumour progression by promoting the inactivation of Src^39^. Thus, we suggest that the temporal-axis genes are associated with or are involved in the transition from a proliferative to a neurogenic state in self-renewing progenitors.

Neurogenesis occurs before E12 in some APs, thus suggesting that neurogenic progenitors are present at E11. However, we failed to detect heterogeneity in the neurogenic/proliferative state of E11 APs populations in both cluster analysis (Fig. 1) and PCA of E11 and E14 APs. PCA of E11+E14 APs (Supplementary Fig. 17) revealed that PC1 represents the temporal difference in APs and that PC2/PC3 represents the cell-cycle phase of the cells, but we failed to find particular functional significance for PC4, PC5 or other components. Thus, PCA of the ‘global gene-expression patterns' at E11 and E14 does not seem to detect subgroups within AP populations.

Because the temporal-axis genes appear to be more sensitive to temporal changes rather than global gene expression, it may be possible to detect heterogeneity in the expression profiles of the top 18 temporal-axis genes. Some of the top 18 temporal-axis genes shown in Fig. 4a indeed exhibited some variation among E11 APs (Fig. 4e). This variation may be correlated with their different division modes. However, we have no data to verify this correlation. The differential gene expression between proliferative APs and neurogenic APs during the transition period should be addressed in future studies. Alternatively, the temporal-axis genes involved in the switch of the division mode might exhibit small changes in their expression levels and thus might not be included in the top 18 temporal-axis genes. In either case, only a minor change in gene expression is likely to occur at the switch, and the differences in gene expression between the time points immediately before and after the switch are quite small. Because the ‘switch' occurs once (and is irreversible) in individual AP lineages and is inherited by the daughter cells, epigenetic modifications in APs are likely to be involved in this switch^26^. Our data regarding the single-cell clonal culture raise the possibility that a limited number of cell-intrinsic factors, which may be included among the temporal-axis genes, play a key role in switching the division mode. In addition, extrinsic factors (and extracellular matrix proteins), which are derived from neighbouring APs may play an important role in coordinating the transitions in global gene-expression patterns in APs *in vivo*.

We also observed changes in Notch signalling status between E11 and E14. This leads us to the interesting possibility that the shift of division mode is related to a ‘threshold of differentiation' in the AP population. Transition of Notch signalling status of APs (Supplementary Fig. 4) may be the consequence of a change in this ‘threshold' during cortical development. This hypothesis predicts that the threshold of Notch activation is relatively lower in E11-type APs, whereas self-renewal requires higher Notch activation at E14-type APs. Further studies are needed to address this issue more fully.

As revealed by transplantation experiments^40^,^41^,^42^, the laminar fates of cortical neurons are, at least in part, determined before they are generated from progenitors. However, the mechanism underlying the shift of progenitor temporal identity, which includes the laminar fate transition of descendant neurons from deep layers to upper layers, has remained largely unknown.

A previous study using the transient inhibition of neuronal production by the expression of NICD suggested that proliferating cortical progenitor cells normally undergo progressive restriction in their laminar fate potential without producing neurons^29^. In this study, we demonstrated that transient cell-cycle arrest (E11–E13) did not interfere with either the transition in the expression of the temporal-axis genes or the laminar fate transition of APs *in vivo*, indicating that cortical progenitors do not require their own cell-cycle progression to promote the temporal progression of the laminar fate potential. In other words, cell-cycle progression does not function as an intrinsic gate keeper of laminar fate change. Notably, we do not exclude the possibility that extrinsic factors from the surrounding cells affect the transition of the temporal character of NICD/p18 co-expressing APs; NICD/p18 co-expressing APs are surrounded by a considerable number of cells that have not been electroporated with the NICD/p18 transgenes and thus undergo normal cell-cycle progression. A recent study proposed that the alternative neuronal specificity of projection neurons and cortico-cortical neurons is determined by a network involving mutual repression between Tbr1, Satb2, Ctip2 and Fezf2 (refs 43, 44, 45). Interestingly, our single-cell transcriptome profiles revealed that both upper-layer and lower-layer marker genes are co-expressed in the same young neurons in the SVZ (Supplementary Fig. 18). It would be interesting to determine how the expression of these marker genes is organized to establish the neuronal identity of individual cells.

In this study, we did not directly address whether the changes in temporal-axis gene expression in APs are related to the progressive restriction of cortical progenitors' potential, such as the laminar fate of their daughter neurons^42^,^46^. Our results indicated that the progression of the temporal-axis genes shares independence from the cell cycle with the transition of laminar fate of the progenies. In addition, major changes in gene expression along the temporal-axis are inherited by IPs from APs. This situation resembles the case of temporal identity genes in *Drosophila* neural progenitor cells, which are expressed in both neuroblasts and ganglion mother cells^47^, indicating that temporal-axis genes satisfy at least one of the criteria that define temporal identity genes (Fig. 3d,e). On the basis of these findings, we argue that the progressive change in temporal-axis gene expression reflects the temporal progression of neural progenitor identity, which involves transitions of both division mode and laminar fate specificity. A recent study revealed that the activation of *Foxg1* is necessary and sufficient to induce deep-layer neurogenesis, followed by a sequential wave of upper-layer neurogenesis^14^. Furthermore, two temporal-axis genes, *Dmrt1a* and *Dmrt3*, are directly suppressed by *Foxg1* (ref. 14). Further studies are needed to examine whether or not these genes are involved in a shift in laminar fate potentials.

The cell-autonomous nature of the laminar fate determination has been suggested by clonal cell culture experiments^46^; these cell-intrinsic changes in progenitor cells are also affected by extrinsic signals^43^. Our *NICD/p18* double-electroporation studies indicated that neither cell-cycle progression nor cell kinesis are necessary in order for APs to change their temporal identity over the course of development. Thus, it is unlikely that cell division works as a ‘timer' in APs to determine the timing of the shift in their temporal identity. This situation is similar to the case in *Drosophila* embryonic neuroblasts^47^,^48^,^49^,^50^, which sequentially express four genes, *hunchback*, *Kruppel*, *pdm1* and *caster*, in that order. Although the *hunchback*→*Kruppel* transition requires neuroblast cytokinesis, the *Kruppel*→*pdm1*→*caster* occurs normally in G2-arrested neuroblasts^49^.

What is the nature of the timer in neural progenitors? In *Drosophila*, the *Kruppel*→*pdm1*→*caster* transitions occur normally in isolated neuroblasts, which give rise to clones consisting of a neuroblast and surrounding descendant cells (which are mutually in contact with each other)^49^, indicating that a ‘lineage-intrinsic' timer underlies neuroblast temporal progression. Similarly, in mammalian systems, clonal culture revealed that the timing of cortical neurogenesis (i.e., sequential generation of Cajal-Retzius neurons, deep-layer neurons and upper-layer neurons) is encoded within lineages of cortical progenitor cells^46^.

In our *in vitro* clonal study, APs were maintained in the one-cell state, allowing us to exclude the effects of contact with surrounding descendant cells. This setup enabled us to examine the actual cell-autonomous mechanism of the lineage-intrinsic timer. We found that the APs in neurospheres, in which mutual effects from surrounding cells are present, exhibited various transitions in the temporal-axis genes as observed *in vivo*, whereas the APs in the one-cell state exhibited a more limited pattern of transition. These findings lead us to propose a model in which the temporal change in APs is partly mediated by a completely cell-intrinsic mechanism, and that extrinsic cues tune the cell-autonomous change in the APs (Fig. 7e). A cascade of transcriptional regulation^48^,^49^, epigenetic modification^1^,^5^,^16^, and subnuclear genome re-organization^51^ may be involved in the actual cell-autonomous changes in AP gene expression. The cell-cycle arrest system used in this study provides a novel paradigm for investigating whether such candidate mechanisms operate cell autonomously.

While in this study the analysis of the single-cell transcriptome was based on microarrays, advanced massive parallel sequencing techniques can process a larger number of single cells to provide high resolution profiles of both coding and non-coding RNAs^52^. Microfluidics applications might also facilitate technically challenging steps *in vitro*, such as Cre induction in single-cell culture. Our findings in this study provide a solid basis for further analyses using such technologies.

## Methods

### Animals

CD1 mice (Crlj:ICR and Slc:ICR) were used throughout the experiments. Tbr2::EGFP (Eomes::EGFP) BAC transgenic mice (Supplementary Fig. 3) were generated by the GENSAT Project^53^, NINDS Contract #N01NS02331 to The Rockefeller University (New York, USA). All animal experiments were performed in accordance with institutional guidelines. To time pregnant mice, the date the vaginal plug was observed was defined as embryonic day (E) 0. The sex of the embryos used was not examined.

### Plasmids

pEF::p18 was generated by cloning mouse *p18* cDNA (NM_007671.2, 227-733), which was cloned by RT-PCR from mouse embryonic brain, into vector pEF-BOS^54^. pEF::loxp-p18-loxp-EGFP-3NLS was constructed by inserting PCR fragments of mouse *p18* cDNA, along with G-CSF polyA from pEF::p18 and EGFP-3NLS from pCAG::EGFP-3NLS^55^, using primers containing the LoxP sequence, into pEF-BOS^54^. pCAG::RFP (mCherry)-3NLS was constructed by swapping EGFP for mCherry cDNA. pCAG-NICD was generated by inserting a fragment containing the intracellular domain of mouse Notch1 (corresponding to amino acids 1,704–2,532 of mouse Notch1) tagged with a FLAG epitope from pME-FNIC^56^ (gift from Dr Ryoichiro Kageyama) into vector pCAG-GS. pCAG::loxp-NICD-IRES-RFP (Strawberry) 3NLS-loxp-EGFP was constructed by inserting LoxP and cDNAs encoding NICD (corresponding to amino acids 1744–2184 of mouse Notch1) and Strawberry-3xNLS into pCAG::EGFP^55^,^57^, and the NICD cDNA was generated from an MGC clone (BC138441) by PCR. pCAG::p27 was generated by cloning mouse *p27* cDNA (BC014296) into pCAG-GS.

### *In utero* electroporation

The abdomen of an anaesthetized pregnant CD1 mouse was dissected with fine scissors and the uterine horns were exposed. A flexible fiber cable (Leica, Wetzlar, Germany) was used for the visualization of embryos in the uterus at E10 and E11 (refs 58, 59). DNA solution was injected into the lateral ventricle using a pulled glass capillary. The head of the embryo in the uterus was then placed between the discs of a forceps-type electrode (disc electrodes of 1 mm for E10–11, CUY560P1; disc electrodes of 3 mm for E13, CUY650P3; NEPA GENE, Chiba, Japan), and electric pulses (50 V for E10–11; 35 V for 13) were discharged four times, resulting in gene transfection into the cerebral wall. The uterus was immediately placed back into the abdominal cavity and the wall and skin of the abdominal cavity were sutured. The final DNA concentrations were as follows: pCAG::EGFP3NLS (0.5 μg μl^−1^)^55^, pCAG::NICD (1.0 μg μl^−1^), and pEF::p18 (1.0 μg μl^−1^) or pCAG::p27 (1.0 μg μl^−1^). In the experiments shown in Fig. 6, a mixture of pCAG::loxp-NICD-IRES-RFP(Strawberry)3NLS-loxp-EGFP (1.0 μg μl^−1^) and pEF::loxp-p18-loxp-EGFP (1.0 μg μl^−1^) was electroporated at E11, and then pCAG::Cre (1.5 μg μl^−1^)^30^ was introduced at E13. As a control, pCAG::EGFP-3NLS (0.5 μg μl^−1^) and pCAG::RFP (mCherry)-3NLS (0.5 μg μl^−1^) were electroporated at E11 and E13, respectively.

We confirmed high co-electroporation efficiency (>98% overlap) in our co-electroporation protocol by electroporation of pCAG::EGFP (0.5 μg μl^−1^) along pEF::mCherry (0.5 μg μl^−1^) (electroporation was performed at E13, and E15 brains were examined; *N*=5 embryos, >220 cells per sample counted). BrdU (100 mg kg^−1^ body weight) or EdU (50 mg kg^−1^ body weight; Life Technologies, Grand Island, USA) was administered by i.p. injection when needed.

### cDNA synthesis from single cerebral cells

Small fragments from the dorso-lateral portion of cerebral wall of a CD1 mouse at E10–13, or small VZ/SVZ fragments of the same portion at E14–16, were digested in 100 μl of 0.25% trypsin/0.5% glucose/PBS for 5 min at 37 °C and triturated. After Hanks solution (Nacalai Tesque, Kyoto, Japan) and trypsin inhibitor (Ovomucoid, Sigma-Aldrich, St Louis, USA) were added, single, isolated cells were manually selected at random by glass capillary under an inverted microscope (Supplementary Fig. 1)^13^. When *in utero* electroporation had been performed previously, EGFP-positive cells were selected on an inverted fluorescence microscope and picked manually. The cell lysis, cDNA synthesis and exponential amplification procedures^21^,^60^ were performed as follows: single cells were transferred to PCR tubes containing 4.5 μl of cell lysis buffer (1 × PCR buffer II, 1.5 mM MgCl_2_ (Applied Biosystems, Foster City, USA), 0.5% NP40 (Roche, Basel, Switzerland), 5 mM DTT (Thermo Fisher Scientific, Waltham, USA), 0.3 U μl^−1^ Prime RNase Inhibitor (Eppendorf, Hamburg, Germany), 0.3 U μl^−1^ RNAguard RNase inhibitor (GE Healthcare, Little Chalfont, UK), 0.2 ng μl^−1^ primer V1[dT]_24_ and 0.05 mM each of dATP, dCTP, dGTP and dTTP) spiked with RNA:poly(A)-tailed *Bacillus subtilis lys, dap, phe* and *thr* RNAs at 1000, 100, 20 and 5 copies per cell, respectively. The sequence of the V1 (dT)_24_ primer was 5′-ATATGGATCCGGCGCGCCGTCGACTTTTTTTTTTTTTTTTTTTTTTTT-3′. After brief centrifugation, cell lysis was performed at 70 °C for 90 s, and the reaction tubes were immediately put on ice for 1 min. After adding 0.3 μl volume of RT mixture (133.3 U μl^−1^ SuperScript III (Thermo Fisher Scientific), 3.33 U μl^−1^ RNAguard RNase Inhibitor and 1.1 μg μl^−1^ T4 gene 32 protein (Roche)), the reaction mixture was incubated at 50 °C for 5 min and heat-inactivated at 70 °C for 10 min. The tubes were immediately put on ice for 1 min, then 1.0 μl of Exonuclease I mixture (1 × Exonuclease I buffer and 0.5 U μl^−1^ Exonuclease I (TAKARA, Tokyo, Japan)) was added to each tube. The reaction mixture was incubated at 37 °C for 30 min, heat-inactivated at 80 °C for 25 min, and was put on ice for 1 min. About 6 μl of terminal deoxynucleotidyl transferase (TdT) mixture (1 × PCR buffer II, 1.5 mM MgCl_2_, 3 mM dATP, 0.1 U μl^−1^ RNaseH and 0.75 U μl^−1^ TdT (Thermo Fisher Scientific)) was added to each tube, and the mixture was incubated at 37 °C for 15 min followed by heat inactivation at 70 °C for 10 min. The synthesized poly(dA)-tailed RT product in each tube (12 μl) was divided into four PCR tubes (3 μl each). Then, 19 μl of PCR mixture I (1 × *ExTaq* buffer, 0.25 mM each of dATP, dCTP, dGTP and dTTP, 0.02 μg μl^−1^ primer V3 (dT)_24_, and 0.05 U μl^−1^ *ExTaq* Hot Start Version (TAKARA)) was added to each tube for the first round of PCR: 95 °C for 3 min, 50 °C for 2 min and 72 °C for 3 min. The sequence of V3 (dT)_24_ was 5′-ATATCTCGAGGGCGCGCCGGATCCTTTTTTTTTTTTTTTTTTTTTTTT-3′. The tubes were immediately put on ice for 1 min, and 19 μl of PCR mixture II was added, with a composition almost the same as that of PCR buffer I but with primer V1 (dT)_24_ replacing primer V3 (dT)_24_. After 20-cycle PCR amplification (95 °C for 30 s, 67 °C for 1 min and 72 °C for 3 min with a 6 s extension per cycle), the amplified cDNA was purified (QIAquick PCR purification kit, Qiagen, Hilden, Germany) and dissolved in 50 μl of buffer EB (10 mM Tris–HCl, pH 8.5). The quality of the amplified cDNA samples was validated by consistent amplification of the spiked RNAs and housekeeping genes (*Gapdh*, *Actb*, *Aldoa* and *Pabpn1*) by qPCR (first quality check).

Some cDNA samples were subjected to another amplification step to add the T7 promoter sequence for the subsequent microarray analysis. A 47.5 μl volume of PCR mixture III (1 × *ExTaq* buffer, 0.25 mM each of dATP, dCTP, dGTP and dTTP, 0.02 μg μl^−1^ primer T7-V1 (5′-GGCCAGTGAATTG TAATACGACTCACTATAGGGAG GCGGATATGGATCCGGC GCGCCGTCGAC-3′), 0.02 μg μl^−1^ primer V3 (dT)_24_ and 0.05 U μl^−1^ *ExTaq* Hot Start Version) was added to each of four 0.2-ml thin-walled PCR tubes containing 2.5 μl of the 20-cycle amplified cDNA. A seven-cycle amplification was then performed according to the following schedule: 95 °C for 5 min 30 s, 64 °C for 1 min and 72 °C for 5 min 18 s for the first cycle; and 95 °C for 30 s, 67 °C for 1 min and 72 °C for 5 min 18 s with an extension of 6 s per cycle for another six cycles. The products were mixed together after the reaction, concentrated by YM-30 or Amicon Ultra-0.5-30K (Merck Millipore, Billerica, USA), then purified with 2% agarose gel electrophoresis to remove by-product DNA shorter than 400 bp. The cDNA was extracted from a gel fragment with a QIAquick Gel Extraction kit (Qiagen) and dissolved in 50 μl of buffer EB. The quality of these second PCR products was again examined by qPCR to detect housekeeping genes and the spiked RNAs (second quality check).

### qPCR analysis

qPCR was performed on cDNAs before addition of the T7 promoter sequence (first quality check or examination of expression levels of genes of interest) and on the products of the second PCR (second quality check) (Supplementary Table 3). Reactions were run on a TP800 real-time PCR system (TAKARA). Ct values were obtained by second derivative maximum method.

### Microarray hybridization and data processing

Among newly generated single-cell-derived cDNAs from murine E11 and E16 cerebral walls (*N*=75 and 62, respectively), 30 (E11) and 28 (E16) samples were selected as representing progenitor populations (Supplementary Table 1) according to marker gene expression determined by qPCR. Single-cell-derived cDNAs from E12 APs (*N*=3) were also selected according to marker gene expression by qPCR among that of E12 cerebral walls (*Ki67*^*+*^*/Ttyh1*^*+*^*/Sox2*^*+*^*/Pax6*^*+*^*/Tbr2*^−^). Single-cell-derived cDNAs from NICD/p18 co-expressing APs (*N*=4, Supplementary Fig. 13), and from APs derived from neurospheres (*N*=3, Supplementary Fig. 15) were also used in the subsequent microarray studies. Subsequently, we obtained their genome-wide transcriptome profiles using DNA microarrays. cDNA samples were subjected to the One-Cycle Target Labelling procedure for biotin labelling by *in vitro* transcription (Affymetrix, Santa Clara, USA), and the resultant cRNA was fragmented and hybridized to the GeneChip Mouse Genome 430 2.0 array (Affymetrix). The microarray image data were processed on a GeneChip Scanner 3000 (Affymetrix), and then analysed using the Affymetrix Microarray Software 5.0 (MAS5.0) algorithm and quantile normalization. A total of 138 cDNA samples were subjected to the GeneChip analysis, and the data were validated by a histogram of the expression values, qcAffy (Affymetrix), RNA degradation, and RLE/NUSE (affyPLM) (third quality check; because of its low quality, one sample (#E11-31i) was excluded from analyses other than that shown in Fig. 1). Linearity between the signal intensity and copy numbers of the original RNAs was validated by monitoring the apparent expression levels of the amplified added RNAs; the signal intensities of RNAs added at >20 copies per cell was proportional to the copy number, as previously reported^13^. Microarrays' signal values of two glial markers, *Gfap* and *S100b*, were very low in all E16 progenitors examined.

### Data analysis

The Over-20 copies probe sets (17192 probe sets) were defined as the probe sets for which at least one sample expressed >20 copies per cell, i.e., a signal intensity ≥870.2 (the median expression level of ‘AFFX-r2-Bs-phe-3_at', the probe set for the spiked RNA *phe*, 20 copies per sample). Because RNAs spiked at ≥20 copies per cell were consistently and proportionally amplified by our method^13^, we used these Over-20 copies probe sets for data analysis. The SigABC genes (114 probe sets) (Fig. 1b–e; Supplementary Figs 13 and 15) were the probe sets that were significantly differently expressed across three typical progenitor groups from E14 (FDR<0.1, ANOVA)^13^.

Cluster analysis of the 128 samples (E11, *N*=30; E14, *N*=70; E16, *N*=28), 104 or 103 samples (E11, *N*=30; E14, *N*=70; NICD/p18, *N*=4 or neurosphere-derived APs, *N*=3) was performed using the GeneChip data from the SigABC genes (Fig. 1b–e; Supplementary Figs 13 and 15). Hierarchical clustering with approximately unbiased (AU) *P* values, computed by multiscale bootstrap resampling to assess the uncertainty in the hierarchical cluster analysis, was performed using the R software package *pvclust* (ref. 61), with the following parameters: distance=‘correlation' and cluster method=‘complete linkage analysis'. AU values indicate how strongly the cluster is supported by data: for example, for a cluster with an AU *P* value>95%, the hypothesis that ‘the cluster does not exist' is rejected with a significance level of 5%.

PCA (principal component analysis) was performed using the R software *prcomp* (scale=FALSE) and the Over-20 copies probe sets data (17,192 probe sets, log values) (Figs 2c and 3a; Supplementary Figs 2, 8, 9, 13, 15 and 17), 21 probe sets for Notch signalling-related genes (Supplementary Fig. 4, log values), or 18 temporal-axis genes [40-Ct] values normalized against *Gapdh* expression levels obtained by qPCR (Fig. 4e; Supplementary Figs 10 and 12). For rotation of PC1/2 to make the median values of NewX of the E11 and E14 APs equal (Fig. 3a,b), the NewX and NewY values were calculated as follows: NewX=cos*θ* × PC1+sin*θ* × PC2; NewY=−sin*θ* × PC1+cos*θ* × PC2; sin*θ*=0.6864510 (*θ*≈43.35°).

To identify probe sets that were significantly differentially expressed across E11, E14 and E16 APs, the GeneChip data (log values) were subjected to ANOVA against the Over-20 copies probe sets on the GeneChip (17,192 probe sets). The FDR was calculated from the two-tailed *P* values using the R software package *qvalue*, and probe sets with FDR<0.1, |Log Fold Change|>2.5 were selected as significantly different probe sets (Fig. 2a,b; Supplementary Data 1).

Functional annotation clustering analysis of ‘PC1 genes', the top 150 probe sets with the highest or lowest PC1 values (Fig. 2c), was performed using DAVID (ref. 25) (Supplementary Table 2).

### RNA *in situ* hybridization

Nonradioactive *in situ* hybridization of frozen sections of E11, E14 or E16 CD1 mouse brain was performed using digoxigenin (Roche)-labelled antisense RNA probes (Fig. 2b; Supplementary Figs 5 and 6). Images were captured on a microscope (BX50, Olympus, Tokyo, Japan) equipped with a CCD digital camera (DP50, Olympus).

### Immunohistochemistry

Brains were fixed in 4% PFA, immersed in 20% sucrose, embedded in OCT compound (Miles, Elkhart, USA), and then frozen and sectioned. Frozen sections were immunostained with mouse anti-BrdU mAb (B2531, 4.4 mg ml^−1^, 1:200, Sigma-Aldrich), chicken anti-EGFP pAb (GFP-1020, 10 mg ml^−1^, 1:2,000, Aves Labs, Tigard, USA), rabbit anti-Trb2 pAb (ab23345, 0.5 mg ml^−1^, 1:300), rabbit anti-Tbr1 pAb (ab31940, 1 mg ml^−1^, 1:300), rabbit anti-Sox2 pAb (ab97959, 1 mg ml^−1^, 1:500) (Abcam, Cambridge, UK), rabbit anti-Pax6 pAb (PRB-278 P, 2 mg ml^−1^, 1:500, Covance, Princeton, USA), rabbit anti-PH3 pAb (06-570, 1 mg ml^−1^, 1:300), rabbit anti-BLBP pAb (ABN14, 1 mg ml^−1^, 1:300) (Merc-Millipore, Darmstadt, Germany), mouse anti-Ki67 mAb (NCL-L-Ki67-MM1, 1:50, Leica Biosystems, Wetzlar, Germany), rabbit anti-RFP pAb (PM005, 1:1,000, MBL, Japan), rabbit anti-Nestin pAb (1:2,000, provided by Dr Yasuoka Tomooka, Tokyo University of Science), mouse anti-Hu mAb (A-21271, 1 mg ml^−1^, 1:200, Life Technologies), mouse anti-βIII tubulin mAb (TuJ1, MMS-435 P, 1 mg ml^−1^, 1:2,000, COVANCE, California, USA), rat anti-RFP mAb (5F8, 1 mg ml^−1^, 1:200, Chromo Tek, Planegg, Germany), or rabbit anti-Cux1 pAb (anti-CDP, sc-13024, 0.2 mg ml^−1^, 1:300, Santa Cruz Biotechnology, Dallas, USA). Secondary antibodies were conjugated to Alexa Fluor 488, 546, or 647 (A11039, A11035, A11081, A11003, A21245, A11056; 2 mg ml^−1^, 1:1,000, Life Technologies). *Antigen*-retrieval by Histo-VT (Nacalai Tesque) or 5 N HCl was performed before staining as needed. EdU was detected using the EdU Alexa Fluor 647 imaging kit (Life Technologies). Immunostained sections were imaged on a laser-scanning confocal microscope (FV1000, Olympus).

### Clonal/neurosphere culture

A mixture of pCAG::loxp-NICD-IRES-RFP-3NLS-loxp-EGFP (1.0 μg μl^−1^) and pEF::loxp-p18-loxp-EGFP-3NLS (1.0 μg μl^−1^) with pCAG::EGFP-3NLS (0.5 μg μl^−1^) was introduced into E10 embryos by *in utero* electroporation. After 1 day, the dorso-lateral portions of cerebral walls including EGFP fluorescence-positive regions were dissected on a stereoscopic fluorescent microscope (Leica Microsystems) and dissociated with 0.25% trypsin/0.2% glucose. After addition of trypsin inhibitor (ovomucoid, Sigma-Aldrich) and DMEM/F12, cells were centrifuged at 900 r.p.m. for 3 min, and then seeded at clonal density (<3 × 10^3^ cells per ml) on 35-mm suspension culture dishes coated with collagen gel (Nitta Gelatin, Japan), which allowed cells to attach very weakly on the bottom of the dish and to be picked following culture. On these dishes, cells were incubated in growth medium (DMEM/F12 including N2, B27 without vitamin A, 10 mM *N*-acetylcysteine, 20 ng ml^−1^ EGF, and 20 ng ml^−1^ FGF2) and cultured at 37 °C in 5% CO_2_. In this culture, although some weakly EGFP^+^ cells formed 2–8-cell clones, strongly EGFP^+^ cells still persisted alone (single-cell clones, Fig. 7b) after 3 days *in vitro* (*div*). The EGFP^++^ single-cell clones that were >400 μm from the other cells were manually selected by using glass capillaries under an inverted fluorescence microscope (Olympus) to generate single-cell cDNAs. As a control experiment, the dorso-lateral portions of cerebral walls from other E11 embryos were also seeded at high density (∼1.0 × 10^5^ cells per ml) in order to form neurospheres. After 3 days *in vitro*, single cells from neurospheres that had been dissociated in 0.25% trypsin/0.2% glucose were manually picked using glass capillaries on an inverted fluorescent microscope (Olympus). Then single-cell cDNAs were generated as described above. Images of live cells were captured on an inverted fluorescence microscope (Olympus) equipped with a CCD digital camera (ORCA-ER, Hamamatsu Photonics, Hamamatsu, Japan; Fig. 7b).

### *In vitro* differentiation of neurospheres

At 3 *div*, 10 μM of BrdU was added to the medium of neurospheres derived from E11 dorso-lateral portion of cerebral cells. After 3 h, the neurospheres were transferred onto polyethylenimine-coated 8-chamber slides (Thermo Fisher Scientific) and cultured in differentiation medium (DMEM/F12 with 5% FBS, N2, B27 without vitamin A, 10 mM *N*-acetylcysteine) at 37 °C in 5% CO_2_. After an additional 5 *div* (8 *div* total), the cells were fixed in 4% PFA and immunostained with anti-BrdU, TuJ1 and anti-Cux1 or anti-Tbr1 antibodies (Supplementary Fig. 16).

### *In vitro* differentiation of NICD/p18-electroporated cells

A mixture of pCAG::loxp-NICD-IRES-RFP-3NLS-loxp-EGFP (1.0 μg μl^−1^) and pEF::loxp-p18-loxp-EGFP-3NLS (1.0 μg μl^−1^) was introduced into E11 embryos by *in utero* electroporation. After 1 day, the dorso-lateral portions of the cerebral walls were dissected and dissociated with 0.25% trypsin/0.2% glucose. After the addition of trypsin inhibitor (ovomucoid, Sigma-Aldrich) and DMEM/F12, the cells were centrifuged at 900 r.p.m. for 3 min, seeded at a low density (3∼6 × 10^3^ cells per ml) on polyethylenimine-coated 8-chamber slides in 300 μl of growth medium (DMEM/F12 with N2, B27 without vitamin A, 10 mM *N*-acetylcysteine, 20 ng ml^−1^ EGF, and 20 ng ml^−1^ FGF2) and cultured at 37 °C in 5% CO_2_. At 2 *div*, 0.03 μl of Adex-CAG-NL-Cre adenovirus (1 × 10^12^ p.f.u. per ml)^62^,^63^ was added to the well, and the medium was changed to differentiation medium (DMEM/F12 with 5% FBS, N2, B27 without vitamin A, 5 ng ml^−1^ EGF, 5 ng ml^−1^ FGF2, and 10 mM *N*-acetylcysteine) after 16 h. After an additional 4 *div* (total 7 *div*), the cells were fixed in 4% PFA and immunostained with anti-GFP, TuJ1 and anti-Cux1 or anti-Tbr1 antibodies (Supplementary Fig. 17).

### Tissue culture with DAPT treatment

Telencephalon tissues prepared from E10 embryos were cultured in hanging drops in growth medium (DMEM/F12 with B27, N2, 5% FBS, 5% horse serum, 10 ng ml^−1^ FGF2 and 20 ng ml^−1^ EGF) containing 10 μM DAPT (gamma-secretase inhibitor *N*-[*N*-(3,5-difluorophenacetyl)-l-alanyl] -*S*-phenylglycine t-butyl ester) (Sigma-Aldrich) or an equal volume of DMSO (Nacalai), at 36 °C with 5% CO_2_ and 30% O_2_. After 10 h, the tissues were fixed in 4% PFA and cryosections were used for immunohistochemistry (Supplementary Fig. 3).

### Statistical analysis

Differences were analysed using Mann–Whitney *U* test with two-tailed *P* values. No statistical methods were used to predetermine sample size, but sample sizes were similar to those described in related previous studies^13^,^59^. No randomization of samples was performed, and no blinding was done. The number of samples examined in each analysis is shown in the legends.

## Additional information

**Accession codes:** Microarray data have been deposited in the GEO database under accession codes: GSE10881 (for E14) and GSE55981 (for E11, E12, E16, NICD/p18, neurosphere-derived APs).

**How to cite this article:** Okamoto, M. *et al*. Cell-cycle-independent transitions in temporal identity of mammalian neural progenitor cells. *Nat. Commun.* 7:11349 doi: 10.1038/ncomms11349 (2016).

## Supplementary Material

Supplementary Information Supplementary Figures 1-18, Supplementary Tables 1-3 and Supplementary References.

Supplementary Data 1 List of genes differentially expressed across E11, E14, and E16 APs (384 probe sets). Probe set IDs were selected (from among 17192 probe sets) by one-way ANOVA from log values of microarray data from N = 23, 33, and 17 single APs from E11, 14, and 16, respectively. FDR < 0.1; E11 vs. E14 or E14 vs. E16 |Log Fold| > 2.5. MS Excel spreadsheet.

Supplementary Data 2 List of genes differentially expressed across E11, E14, and E16 IPs (312 probe sets). Probe set IDs were selected (from among 17,192 probe sets) by one-way ANOVA from log values of microarray data from N = 3, 23, and 10 single IPs from E11, 14, and 16, respectively. FDR < 0.1; E11 vs. E14 or E14 vs. E16 |Log Fold| > 2.5. Since there are a few E11 IP samples (N = 3), many temporal-axis genes in Fig. 3 are not included in this list because of their FDR values. MS Excel spreadsheet.
