# Supplementary material for: Cell-cycle-independent transitions in temporal identity of mammalian neural progenitor cells
Source: Nat Commun. 2016 Apr 20;7:11349. doi: 10.1038/ncomms11349 (PMC4842982; doi:10.1038/ncomms11349)
Supplement: Supplementary Information — Supplementary Figures 1-18, Supplementary Tables 1-3 and Supplementary References. [file ncomms11349-s1.pdf]

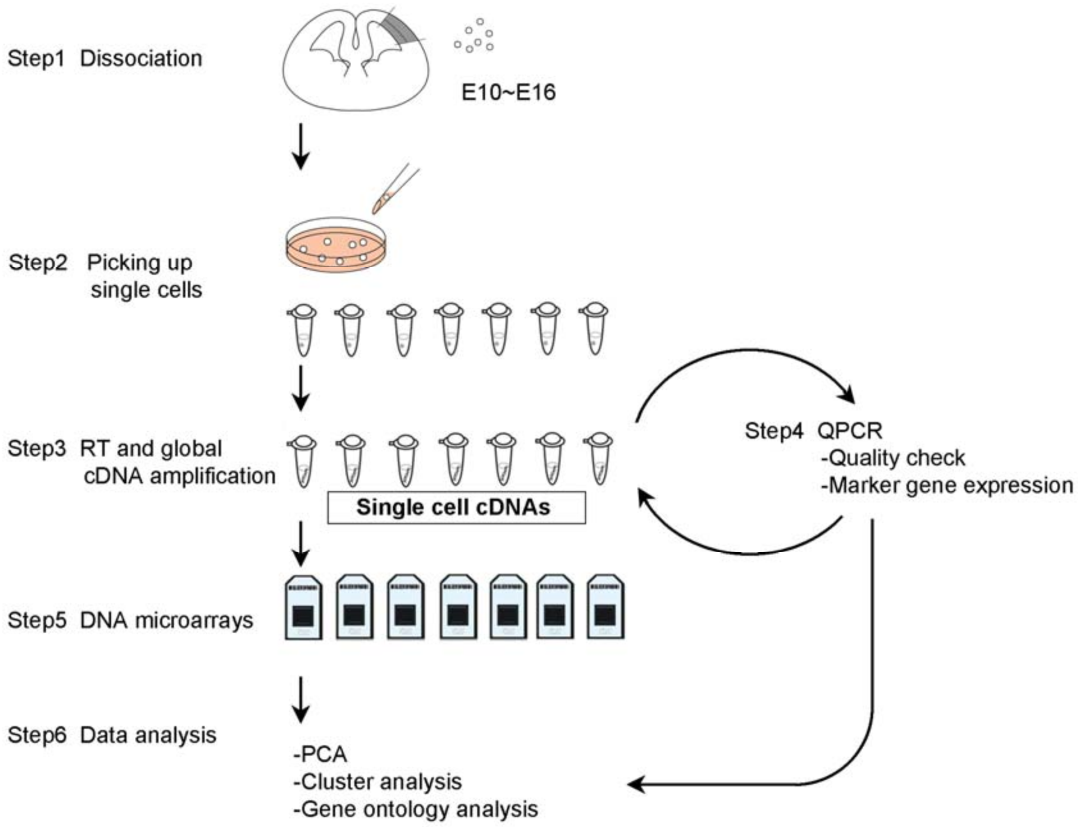

Supplementary Figure 1. Experimental design.

Step1: The dorso-lateral portion of the cerebral wall (E10–E13) or VZ/SVZ fragment of the same portion of the cerebral wall (E14–E16) was dissected and dissociated by trypsin.

Step2: Single cells were manually selected at random by using a glass capillary.

Step3: After cell lysis, single cell cDNAs were generated by reverse transcription and global amplification.

Step4: The quality of cDNAs and marker gene expression were examined by QPCR.

Step5: Selected samples (Supplementary Table 1) were analyzed by DNA microarrays (E11, E12, E14, and E16).

Step6: Data from microarrays or QPCR were analyzed by PCA, cluster analysis or gene ontology analysis.

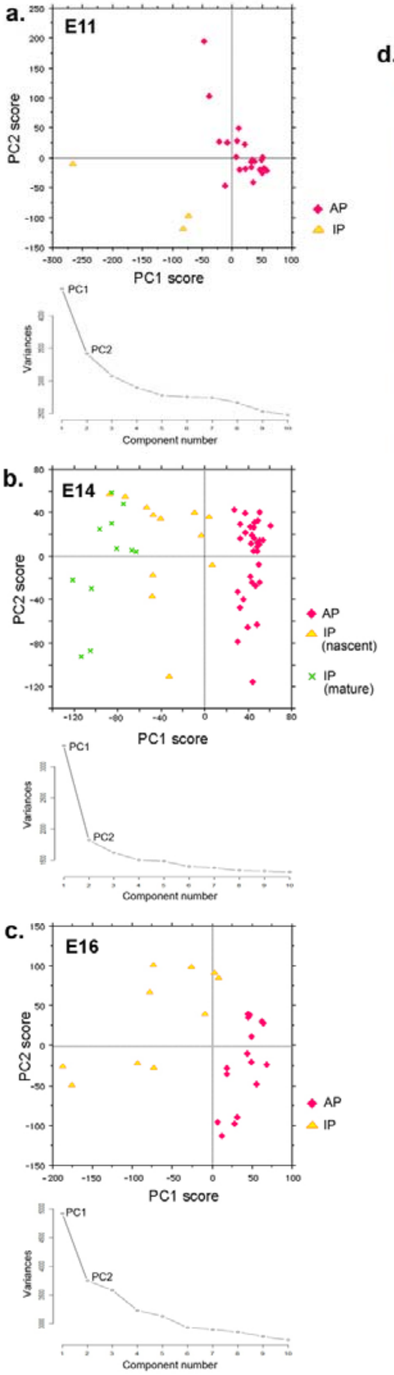

d.

E11	PC1		E14	PC1		E16	PC1	
Probeset ID	Gene Symbol	PC1	ProbesetID	Gene Symbol	PC1	ProbesetID	Gene Symbol	PC1
1433643_at	Cacna2d1	-2.88×10^{-2}	1418047_at	Neurod6	-4.94×10^{-2}	1436729_at	Afap1	-3.49×10^{-2}
1455865_at	Insm1	-2.83×10^{-2}	1436729_at	Afap1	-4.69×10^{-2}	1418047_at	Neurod6	-3.36×10^{-2}
1426001_at	Eomes	-2.83×10^{-2}	1434531_at	Mgat5b	-4.59×10^{-2}	1421882_a_at	Elavl2	-3.24×10^{-2}
1438551_at	Neurog1	-2.81×10^{-2}	1452894_at	Elavl4	-4.50×10^{-2}	1415978_at	Tubb3	-3.20×10^{-2}
1436888_at	Nhlh2	-2.79×10^{-2}	1426001_at	Eomes	-4.36×10^{-2}	1421883_at	Elavl2	-3.16×10^{-2}
1452646_at	Trp53inp2	-2.67×10^{-2}	1421883_at	Elavl2	-4.35×10^{-2}	1433988_s_at	C230098O21Rik	-3.15×10^{-2}
1435172_at	Eomes	-2.66×10^{-2}	1416107_at	Nsg2	-4.33×10^{-2}	1436050_x_at	Hes6	-3.10×10^{-2}
1426412_at	Neurod1	-2.62×10^{-2}	1435172_at	Eomes	-4.30×10^{-2}	1452894_at	Elavl4	-3.03×10^{-2}
1425575_at	Epha3	-2.60×10^{-2}	1456387_at	Nol4	-4.25×10^{-2}	1455324_at	Plcxd2	-2.96×10^{-2}
1452393_at	Akna	-2.58×10^{-2}	1453851_a_at	Gadd45g	-4.22×10^{-2}	1449660_s_at	Coro1c	-2.90×10^{-2}
1425784_a_at	Olfm1	-2.55×10^{-2}	1418139_at	Dcx	-4.10×10^{-2}	1452779_at	3110006E14Rik	-2.88×10^{-2}
1453851_a_at	Gadd45g	-2.54×10^{-2}	1455426_at	Epha3	-4.06×10^{-2}	1437357_at	Ythdc2	-2.86×10^{-2}
1426413_at	Neurod1	-2.53×10^{-2}	1421882_a_at	Elavl2	-4.03×10^{-2}	1440286_at	Fbxl16	-2.86×10^{-2}
1422256_at	Sstr2	-2.51×10^{-2}	1416992_at	Mfng	-4.02×10^{-2}	1434531_at	Mgat5b	-2.81×10^{-2}
1450099_a_at	Gba	-2.50×10^{-2}	1452646_at	Trp53inp2	-3.87×10^{-2}	1433571_at	Serinc5	-2.78×10^{-2}
1429390_at	Acpl2	-2.49×10^{-2}	1428393_at	Nrn1	-3.86×10^{-2}	1438321_x_at	4930504E06Rik	-2.77×10^{-2}
1426484_at	Ubxd2	-2.46×10^{-2}	1455796_x_at	Olfm1	-3.77×10^{-2}	1423328_at	Gdap1	-2.76×10^{-2}
1448943_at	Nrp1	-2.46×10^{-2}	1460038_at	Pou3f1	-3.73×10^{-2}	1416992_at	Mfng	-2.75×10^{-2}
1435941_at	Rhbdl3	-2.43×10^{-2}	1422773_at	Myt1	-3.72×10^{-2}	1429175_at	Tmem178	-2.75×10^{-2}
1455123_at	St18	-2.40×10^{-2}	1433988_s_at	C230098O21Rik	-3.70×10^{-2}	1454961_at	Synj1	-2.73×10^{-2}

Probeset ID	Gene Symbol	PC1	ProbesetID	Gene Symbol	PC1	ProbesetID	Gene Symbol	PC1
1422851_at	Hmga2	3.27×10^{-2}	1437347_at	Ednrb	5.18×10^{-2}	1422694_at	Ttyh1	3.55×10^{-2}
1437279_x_at	Sdc1	3.21×10^{-2}	1438511_a_at	1190002H23Rik	4.91×10^{-2}	1448162_at	Vcam1	3.52×10^{-2}
1436420_a_at	Ipo4	3.14×10^{-2}	1452114_s_at	Igfbp5	4.90×10^{-2}	1418969_at	Skp2	3.48×10^{-2}
1428333_at	Arxes2	3.13×10^{-2}	1423259_at	Id4	4.55×10^{-2}	1437687_x_at	Fkbp9	3.36×10^{-2}
1455034_at	Nr4a2	2.93×10^{-2}	1422694_at	Ttyh1	4.35×10^{-2}	1460247_a_at	Skp2	3.30×10^{-2}
1416345_at	Timm8a1	2.90×10^{-2}	1450042_at	Arx	4.07×10^{-2}	1448181_at	Klf15	3.23×10^{-2}
1423259_at	Id4	2.90×10^{-2}	1460587_at	B230215L15Rik	3.92×10^{-2}	1453006_at	Fgfbp3	3.19×10^{-2}
1451012_a_at	Csda	2.87×10^{-2}	1448162_at	Vcam1	3.87×10^{-2}	1427347_s_at	Tubb2a	3.14×10^{-2}
1417140_a_at	Ptpn2	2.76×10^{-2}	1450644_at	Zfp36l1	3.82×10^{-2}	1437347_at	Ednrb	3.13×10^{-2}
1422694_at	Ttyh1	2.74×10^{-2}	1449065_at	Acot1	3.79×10^{-2}	1419838_s_at	Plk4	3.09×10^{-2}
1422844_a_at	Wdr77	2.73×10^{-2}	1455556_at	Notch2	3.75×10^{-2}	1436750_a_at	Oxct1	3.02×10^{-2}
1419462_s_at	Gtl3	2.72×10^{-2}	1451071_a_at	Atp1a1	3.75×10^{-2}	1451835_at	Sox21	2.98×10^{-2}
1452896_at	Gtl3	2.72×10^{-2}	1448694_at	Jun	3.73×10^{-2}	1448754_at	Rbp1	2.95×10^{-2}
1455643_s_at	Tsr1	2.70×10^{-2}	1435578_s_at	Dab1	3.72×10^{-2}	1451188_at	Wdr26	2.92×10^{-2}
1450780_s_at	Hmga2	2.70×10^{-2}	1427019_at	Ptprz1	3.69×10^{-2}	1437689_x_at	Clu	2.91×10^{-2}
1435800_a_at	Csda	2.69×10^{-2}	1438729_at	BB176347	3.67×10^{-2}	1437671_x_at	Prss23	2.89×10^{-2}
1435578_s_at	Dab1	2.67×10^{-2}	1434264_at	Ank2	3.67×10^{-2}	1448392_at	LOC100046740	2.87×10^{-2}
1449576_at	Eif1ay	2.64×10^{-2}	1418003_at	1190002H23Rik	3.66×10^{-2}	1424292_at	Depdc1a	2.87×10^{-2}
1460677_at	Spats2	2.62×10^{-2}	1452284_at	Ptprz1	3.64×10^{-2}	1436761_s_at	1200015N20Rik	2.83×10^{-2}
1435577_at	Dab1	2.58×10^{-2}	1421344_a_at	Jub	3.61×10^{-2}	1429019_s_at	Pon2	2.80×10^{-2}

E11	PC2		E14	PC2		E16	PC2	
Probeset ID	Gene Symbol	PC2	ProbesetID	Gene Symbol	PC2	ProbesetID	Gene Symbol	PC2
1448026_at	Chd7	-3.23×10^{-2}	1452881_at	Gins2	-3.27×10^{-2}	1416329_at	Cyfip1	-3.20×10^{-2}
1415908_at	Tspyl1	-3.22×10^{-2}	1423809_at	Tcf19	-3.07×10^{-2}	1422538_at	Extl2	-3.09×10^{-2}
1426840_at	Ythdf3	-3.11×10^{-2}	1439249_at	LOC100044766	-2.89×10^{-2}	1435148_at	Atp1b2	-2.81×10^{-2}
1417372_a_at	Peli1	-3.07×10^{-2}	1433509_s_at	Reep1	-2.84×10^{-2}	1456509_at	1110032F04Rik	-2.71×10^{-2}
1449519_at	Gadd45a	-3.04×10^{-2}	1420745_a_at	Ccndbp1	-2.79×10^{-2}	1437689_x_at	Clu	-2.68×10^{-2}
1433864_at	Lrp12	-3.03×10^{-2}	1424694_at	2010011I20Rik	-2.78×10^{-2}	1438155_x_at	Pigo	-2.64×10^{-2}
1450664_at	Gabpa	-2.91×10^{-2}	1456542_s_at	Qrsl1	-2.74×10^{-2}	1423889_at	EG434402	-2.62×10^{-2}
1436600_at	Tox3	-2.91×10^{-2}	1424143_a_at	Cdt1	-2.73×10^{-2}	1452881_at	Gins2	-2.62×10^{-2}
1437327_x_at	Enoph1	-2.90×10^{-2}	1424144_at	Cdt1	-2.69×10^{-2}	1456377_x_at	Limd2	-2.57×10^{-2}
1418915_at	Mmachc	-2.88×10^{-2}	1455558_at	Gm114	-2.67×10^{-2}	1454939_at	---	-2.56×10^{-2}
1455121_at	Lcor	-2.82×10^{-2}	1417868_a_at	Ctsz	-2.57×10^{-2}	1436761_s_at	1200015N20Rik	-2.55×10^{-2}
1420505_a_at	Stxbp1	-2.80×10^{-2}	1455970_at	---	-2.49×10^{-2}	1437987_at	---	-2.53×10^{-2}
1433570_s_at	Mak10	-2.79×10^{-2}	1436175_at	Atxn7	-2.41×10^{-2}	1419490_at	AW049604	-2.48×10^{-2}
1424076_at	Gdpd1	-2.77×10^{-2}	1452127_a_at	Ptpn13	-2.39×10^{-2}	1460324_at	Dnmt3a	-2.46×10^{-2}
1436833_x_at	Ttll1	-2.71×10^{-2}	1428713_s_at	Gins2	-2.38×10^{-2}	1417600_at	Slc15a2	-2.45×10^{-2}
1436390_a_at	Clcc1	-2.67×10^{-2}	1416950_at	Tnfaip8	-2.38×10^{-2}	1451377_a_at	Aaas	-2.43×10^{-2}
1423857_at	Mrpl30	-2.66×10^{-2}	1418436_at	Stx7	-2.33×10^{-2}	1454860_x_at	Dad1	-2.41×10^{-2}
1449192_at	Atf7ip	-2.65×10^{-2}	1423293_at	Rpa1	-2.29×10^{-2}	1418626_a_at	Clu	-2.41×10^{-2}
1423132_a_at	Nsa2	-2.60×10^{-2}	1452733_at	Pank2	-2.26×10^{-2}	1437458_x_at	Clu	-2.40×10^{-2}
1421015_s_at	Pole3	-2.60×10^{-2}	1418507_s_at	Socs2	-2.21×10^{-2}	1436540_at	6720427I07Rik	-2.40×10^{-2}

Probeset ID	Gene Symbol	PC2	ProbesetID	Gene Symbol	PC2	ProbesetID	Gene Symbol	PC2
1448754_at	Rbp1	3.06×10^{-2}	1456077_x_at	Cdc25c	5.11×10^{-2}	1456077_x_at	Cdc25c	3.86×10^{-2}
1426607_at	EG633640	2.99×10^{-2}	1433807_at	6720463M24Rik	4.61×10^{-2}	1433807_at	6720463M24Rik	3.77×10^{-2}
1435467_at	Fgd6	2.92×10^{-2}	1416076_at	Ccnb1	4.60×10^{-2}	1424591_at	5830433M19Rik	3.73×10^{-2}
1435926_at	Chml	2.83×10^{-2}	1452242_at	Cep55	4.56×10^{-2}	1438434_at	Arhgap11a	3.46×10^{-2}
1419417_at	Vegfc	2.75×10^{-2}	1455730_at	Dlg7	4.55×10^{-2}	1437370_at	Sgol2	3.45×10^{-2}
1456574_at	Zfp800	2.64×10^{-2}	1448205_at	Ccnb1	4.50×10^{-2}	1437133_x_at	Akr1b3	3.42×10^{-2}
1439766_x_at	Vegfc	2.62×10^{-2}	1424105_a_at	Pttg1	4.46×10^{-2}	1456590_x_at	Akr1b3	3.42×10^{-2}
1451171_at	2310008H04Rik	2.51×10^{-2}	1436738_at	Pif1	4.45×10^{-2}	1434911_s_at	Arhgap19	3.37×10^{-2}
1434586_a_at	Ptdss2	2.49×10^{-2}	1437580_s_at	Nek2	4.44×10^{-2}	1426256_at	Timm17a	3.28×10^{-2}
1416498_at	Ppic	2.47×10^{-2}	1422252_a_at	Cdc25c	4.37×10^{-2}	1434789_at	Depdc1b	3.23×10^{-2}
1418716_at	Mrps25	2.46×10^{-2}	1442280_at	D2Ertd750e	4.31×10^{-2}	1455608_at	Sclt1	3.16×10^{-2}
1429065_at	Ikbip	2.45×10^{-2}	1438149_at	Pif1	4.25×10^{-2}	1452242_at	Cep55	3.15×10^{-2}
1452676_a_at	Pnpt1	2.44×10^{-2}	1453416_at	Gas2l3	4.14×10^{-2}	1437611_x_at	Kif2c	3.14×10^{-2}
1422616_s_at	Wdr54	2.43×10^{-2}	1439394_x_at	Cdc20	4.00×10^{-2}	1448205_at	Ccnb1	3.12×10^{-2}
1416743_at	Uap1	2.40×10^{-2}	1429326_at	Cenpl	3.98×10^{-2}	1436725_at	E130306D19Rik	3.11×10^{-2}
1453006_at	Fgfbp3	2.38×10^{-2}	1450842_a_at	Cenpa	3.97×10^{-2}	1451536_at	Mtfr1	3.10×10^{-2}
1416629_at	Slc1a5	2.37×10^{-2}	1450156_a_at	Hmmr	3.90×10^{-2}	1422252_a_at	Cdc25c	3.10×10^{-2}
1417743_at	Clk2	2.37×10^{-2}	1419943_s_at	Ccnb1	3.88×10^{-2}	1453107_s_at	Foxm1	3.09×10^{-2}
1426936_at	BC005512	2.37×10^{-2}	1433543_at	Anln	3.85×10^{-2}	1428052_a_at	Zmym1	3.08×10^{-2}
1451989_a_at	Mapre2	2.35×10^{-2}	1450920_at	Ccnb2	3.81×10^{-2}	1416076_at	Ccnb1	3.07×10^{-2}

Supplementary Figure 2. Unsupervised PCA reveals that the ‘AP vs. IP axis’ mostly reflects the variety of progenitor cells at each stage.

(a–c) PCA was performed on progenitor samples at each stage, using all available probe set data (17192 probe sets; a, E11, N = 26; b, E14, N = 56; c, E16, N = 27). Each symbol indicates one cell, and the categorization of the symbols (AP or IP) is based on the classification from the results shown in Fig. 1b–d. Principal component 1 (PC1) defined the axis along which the variance in gene expression was largest among the tested cells. At E14, the PC1 axis clearly depicted the differentiation state of progenitors; APs, nascent IPs, and mature IPs were plotted as different groups along the axis in that order (b). This order was reproduced in the case of E11 (a) and E16 cells (c). Scree plots were also shown.

Proportion of variances: (a) 0.071 for PC1, 0.055 for PC2, (b) 0.051 for PC1, 0.028 for PC2, and (c) 0.070 for PC1, 0.053 for PC2.

(d) Genes that contribute highly to PC1 and PC2 of E11, E14, and E16 progenitors.

Lists show the top 20 genes that contribute most strongly to PC1 or PC2, positively or negatively, based on the results of the PCA shown in (a–c). According to the functional annotation clustering analysis, genes that contribute highly to PC1 at E11, E14, and E16 were significantly related to 'differentiation' or 'neuron differentiation' (N = 40, 40, and 100 probe sets for each) ($p = 1.5 \times 10^{-4}$, 6.6×10^{-5}, 1.8×10^{-4}, respectively), and those contributing to PC2 at E14 or E16 (N = 40 probe sets each) were significantly related to the 'cell cycle' ($p = 1.5 \times 10^{-13}$ and $p = 5.9 \times 10^{-4}$), suggesting that the PC2 scores at E14 and E16 reflected the cell-cycle phase of the tested cells.

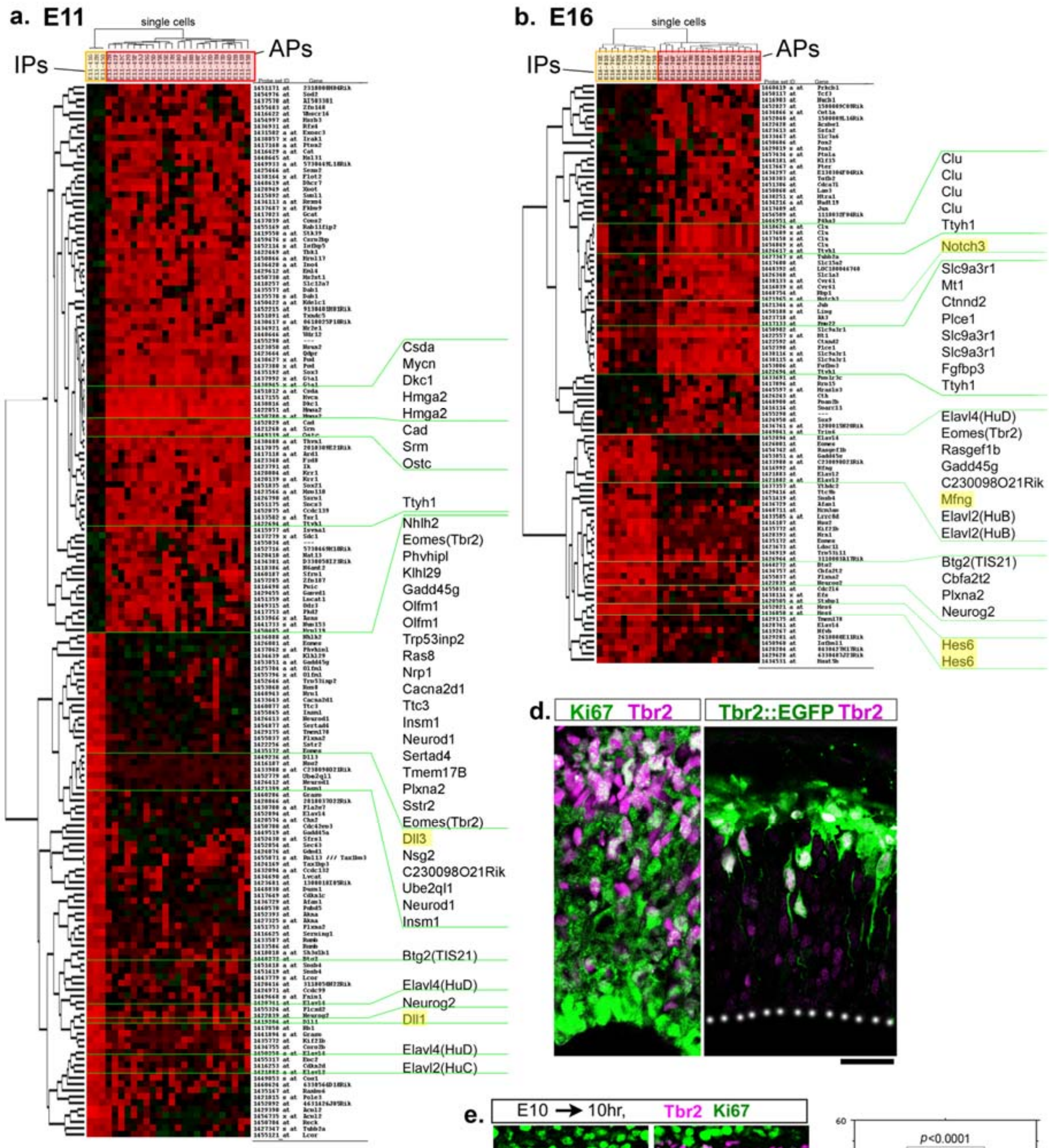

c. E11 APs vs IPs

Signaling pathway	*p*-values
Gadd45 signaling	0.0121
Cell cycle regulation by BTG family proteins	0.0344
Inhibition of matrix metalloproteinases	0.0344
Notch signaling	0.0384

E16 APs vs IPs

Signaling pathway	*p* -values
14-3-3-mediated signaling	0.00079
Epihelial adherens junction signaling	0.00184
Wnt/β-catenin signaling	0.00306
Notch signaling	0.00802

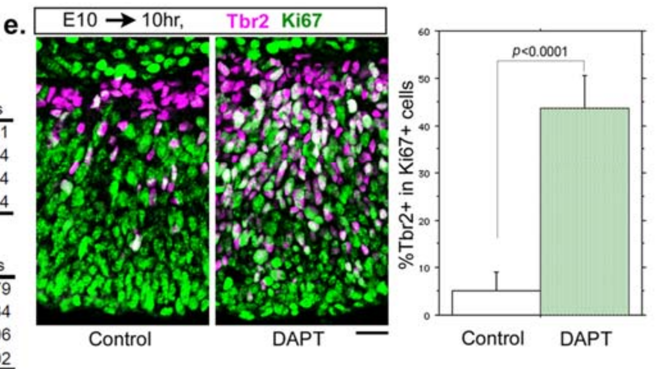

Supplementary Figure 3. Differentially expressed genes between AP and IP at E11 and E16.

(a, b) Two-way clustering analysis using the significantly differently expressed genes between APs and IPs at E11 (N = 23 APs vs. N = 3 IPs, 167 probe sets were selected [from 17192 total probe sets] by Welch's *t*-test according to the

following criteria: |Log Fold| > 4.0, FDR < 0.1), or at E16 (N = 17 APs vs. N = 10 IPs, 96 probe sets were selected [from 17192 total probe sets] by Welch's *t*-test according to the following criteria: |Log Fold| > 3.0, FDR < 0.1). Many of the IP-specific genes are common among E11, E14 [1], and E16.

(c) Pathway analysis (Ingenuity Pathway Analysis, IPA) of differentially expressed genes between APs and IPs at E11 (a) or E16 (b) revealed that the Notch-signaling pathway was the significantly different canonical signaling pathways in both stages.

(d) $Tbr2^+$ cells commit to become IPs in the E11 cerebral wall. (left) Double immunostaining of the E11 cerebral wall with antibodies against Ki67 (green) and Tbr2 (magenta). $Ki67^+$ progenitor cells include both $Tbr2^−$ and $Tbr2^+$ cells. (right) EGFP (green) and Tbr2 (magenta) expression in the cerebral wall of the E11 Tbr2::EGFP BAC transgenic mouse [2, 3]. Because EGFP is a relatively stable protein, EGFP could be used to short-chase the fate of $Tbr2^+$ cells [1]. There were no $EGFP^+$/$Tbr2^−$ cells in the VZ, suggesting that once cells express Tbr2, they never return to the apical surface as $Tbr2^−$ apical progenitor cells. Dotted line, apical surface.

(e) Inhibition of Notch signaling converts the E10 APs to the IPs. 10 μM of Notch inhibitor DAPT or control DMSO was added into the medium of the E10 telencephalon tissue culture for 10 hours. The frequency of $Tbr2^+$ cells in $Ki67^+$ progenitors was significantly increased by DAPT treatment ($p < 0.0001$, Mann-Whitney U test, means ± s.d.). Bar, 30 μm.

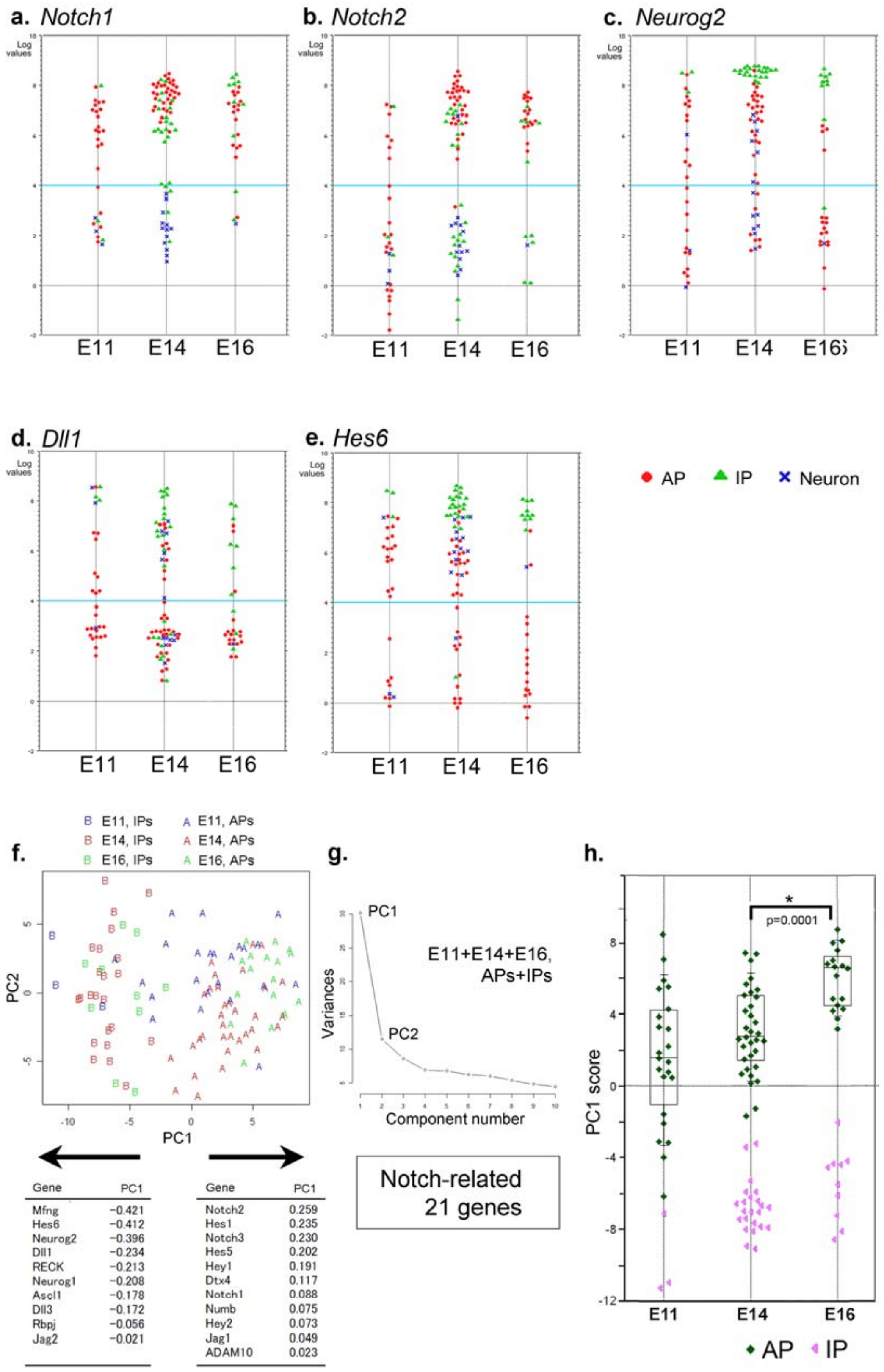

Gene	PC1
Mfng	−0.421
Hes6	−0.412
Neurog2	−0.396
Dll1	−0.234
RECK	−0.213
Neurog1	−0.208
Ascl1	−0.178
Dll3	−0.172
Rbpj	−0.056
Jag2	−0.021

Gene	PC1
Notch2	0.259
Hes1	0.235
Notch3	0.230
Hes5	0.202
Hey1	0.191
Dtx4	0.117
Notch1	0.088
Numb	0.075
Hey2	0.073
Jag1	0.049
ADAM10	0.023

Supplementary Figure 4. Cell-to-cell variation of Notch signaling–related gene expression in APs changes over the course of cortical development.

(a–e) *Notch1, Notch2, Neurog2, Dll1*, and *Hes6* expression levels in the cortical cells. Scatter diagrams of microarray

data from 30, 70, and 28 single-cell cDNAs at E11, E14, and E16, respectively. Each symbol indicates one cell, and axes are in the natural logarithmic scale.

(f-h) To describe how the variation of Notch signaling status among cortical progenitor cells changes during cortical development, PCA on all progenitor cells (E11 + E14 + E16, APs + IPs, N = 109) was performed, using the expression values of the 21 genes related to Notch signaling (f).

(f) Score plot of the PC1 and PC2 values for all tested progenitor cells. Each symbol represents one progenitor cell. The contribution of each gene to the PC1 is shown in the bottom tables.

(g) The scree plot of (f) indicated that PC1 outstandingly represented the gene expression variation, and validated the PC1 score (h) as an indicator of 'Notch signaling status'. Proportion of variance, 0.2616 (PC1),

(h) Scatter diagram of the PC1 scores for E11, E14, and E16 progenitor cells. The PC1 scores of E11 APs were highly variable, ranging from negative to positive values. As development proceeded, this variation became smaller (*F*-test, E11 vs. E14, $p = 0.0209$; E11 vs. E16, $p = 0.0014$), and the mean PC1 score increased, as in APs (Mann-Whitney *U* test, E14 vs. E16, $p = 0.0001$). Each symbol represents one progenitor cell.

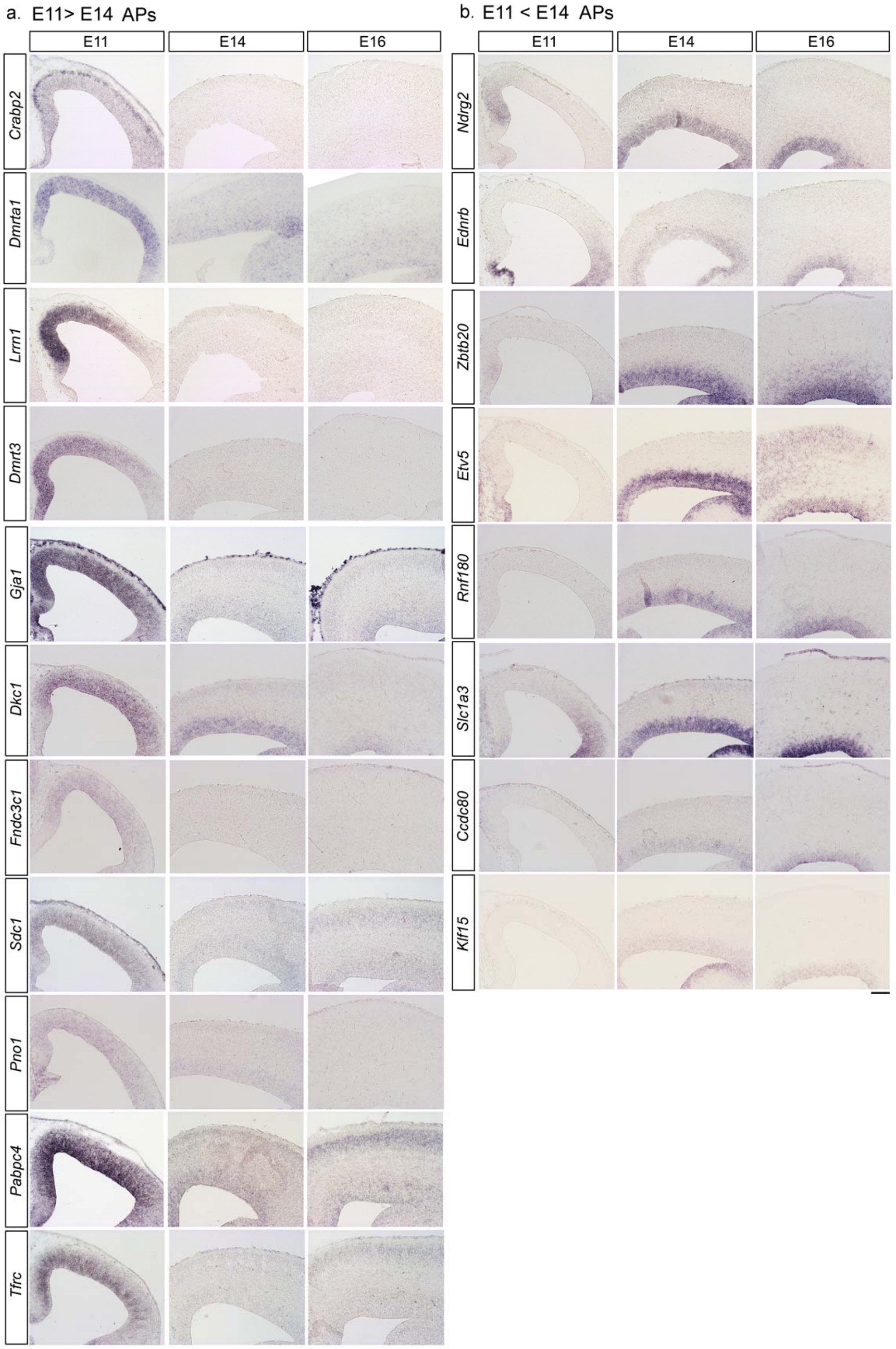

Supplementary Figure 5. *In situ* expression pattern of genes whose expression levels change between E11 and E14 in APs.

Temporal changes in genes selected from single-cell gene-expression profiles of APs (Fig. 2a, Supplementary Data 1) were also observed by *in situ* hybridization of E11, E14, and E16 mouse cerebral wall. Results from E11 > E14 genes **(a)** and E11 < E14 genes **(b)** are shown. Bar, 100μm.

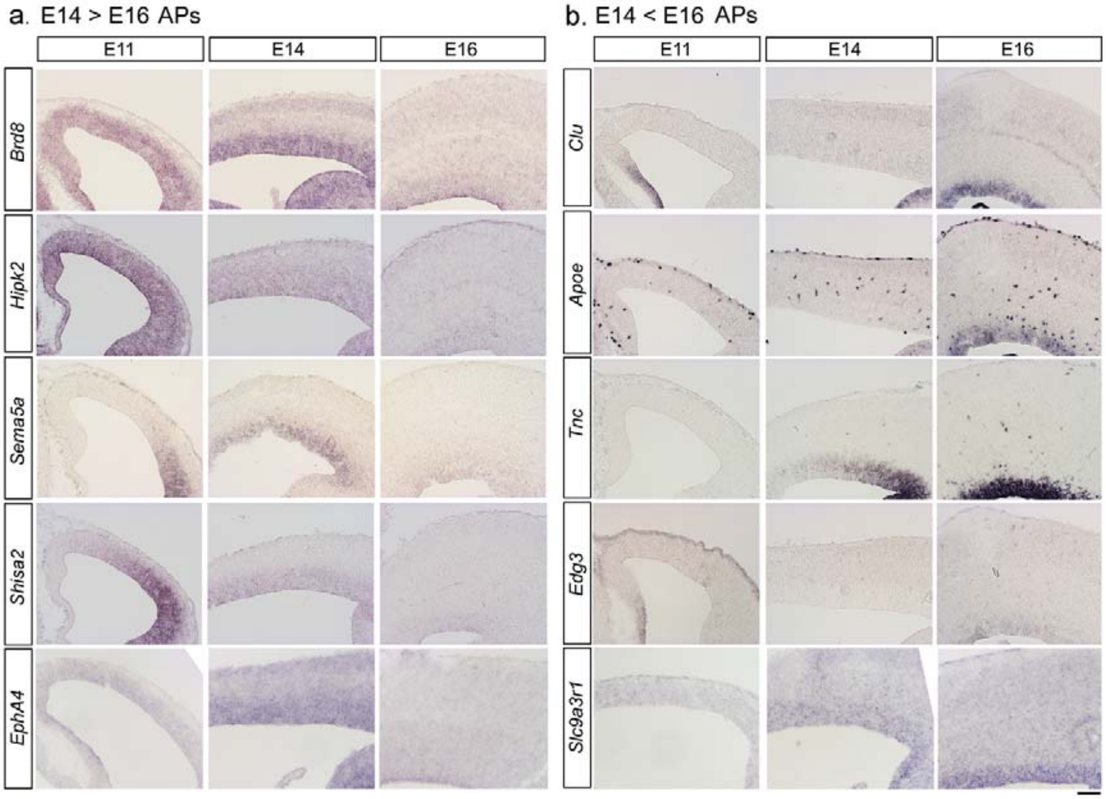

Supplementary Figure 6. *In situ* expression of genes whose expression levels change between E14 and E16 in APs. Temporal changes in genes selected from single-cell gene-expression profiles of APs (Fig. 2a, Supplementary Data 1) were also observed by *in situ* hybridization of E11, E14, and E16 mouse cerebral wall. Results from E14 > E16 genes **(a)** and E14 < E16 genes **(b)** are shown. Bar, 100µm.

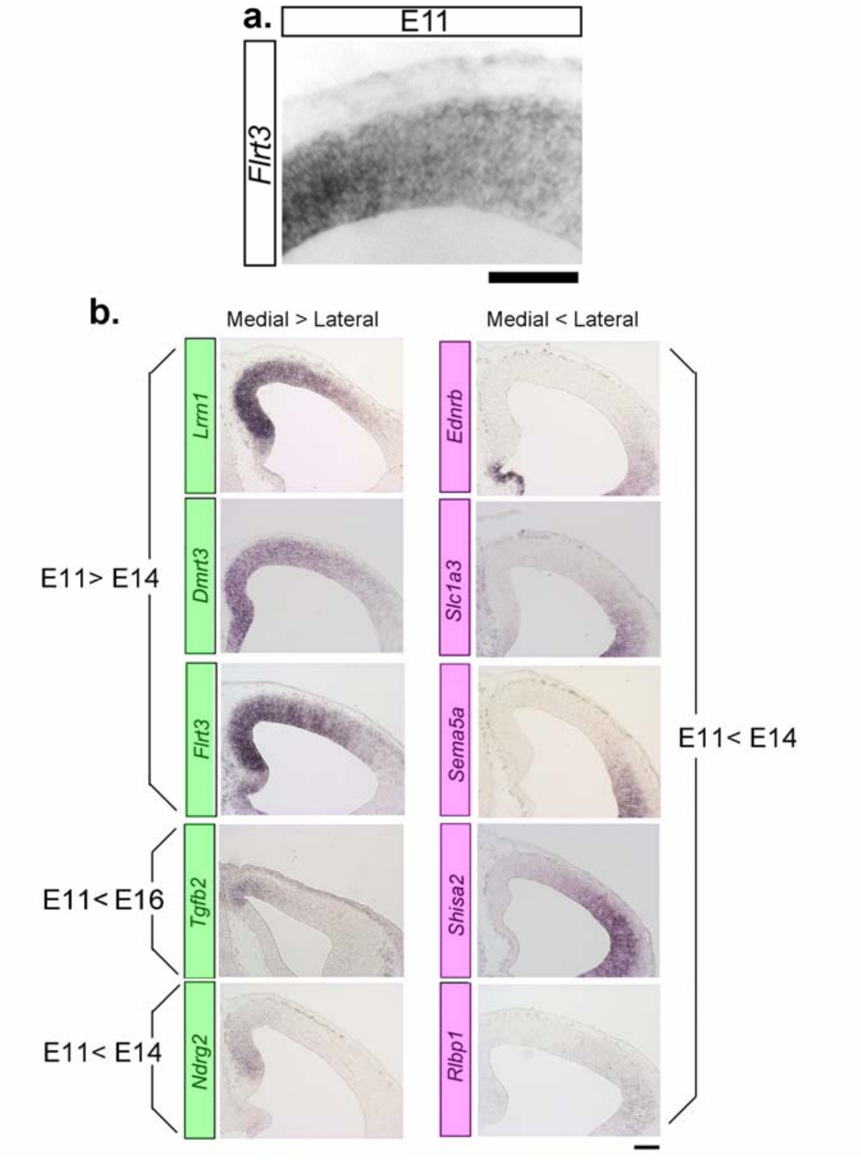

Supplementary Figure 7. Mediolateral gradient of gene expression.

(a) In situ expression of *Flrt3* revealed cell-to-cell variations of its expression level in a mediolateral gradient.

(b) Examples of genes that exhibit a 'medial > lateral' gradient pattern (green) or 'medial < lateral' gradient pattern (magenta). The 'medial > lateral' genes exhibited an 'E11 > E14' trend, and the 'medial < lateral' genes exhibited an 'E11 < E14' trend, consistently with previous reports showing that cortical neurogenesis proceeds in the lateral to medial direction [4, 5]. Bar, 100μm.

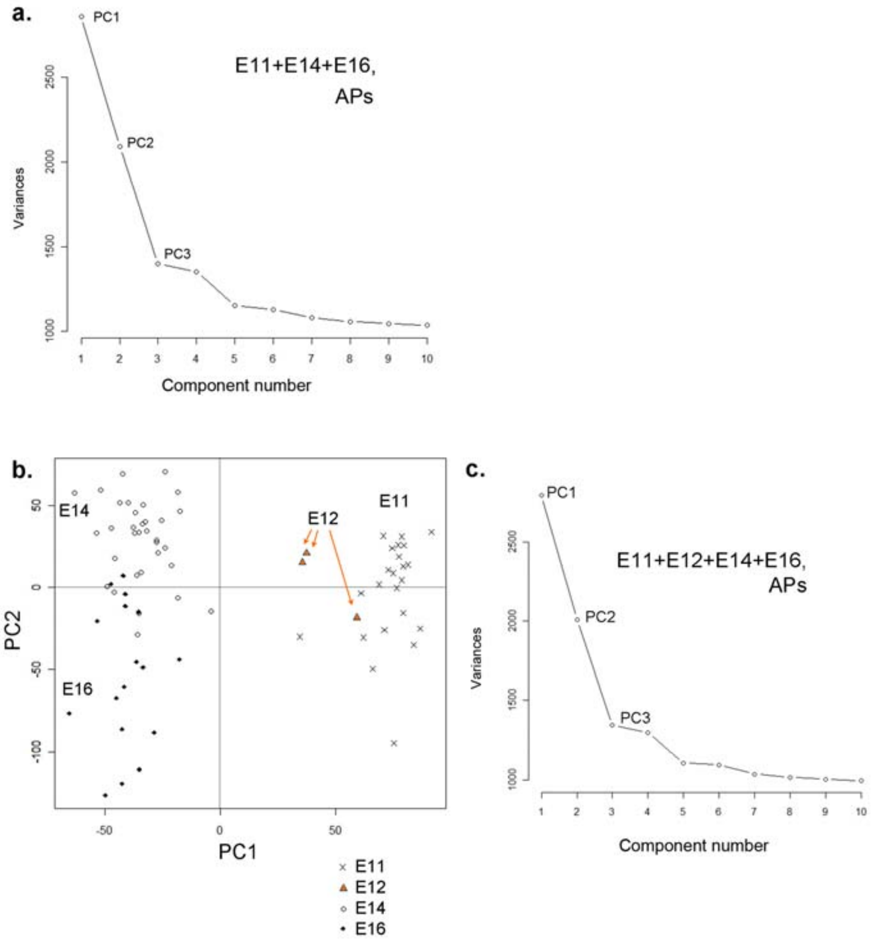

Supplementary Figure 8. PCA on AP samples derived from different developmental stages.

(a) Scree plot of PCA for all APs of E11, E14 and E16 in Fig2c.

(b) E12 APs show the intermediate state between E11 and E14 APs in their global gene expression pattern. PCA was performed on E12 AP samples together with E11, E14, and E16 AP samples, using all available probe set data (17192 probe sets; E11, N = 26; E12, N = 3; E14, N = 56; E16, N = 27). Each symbol indicates one cell.

(c) Scree plot of (b). Proportion of variances: 0.0430 for PC1 and 0.0309 for PC2.

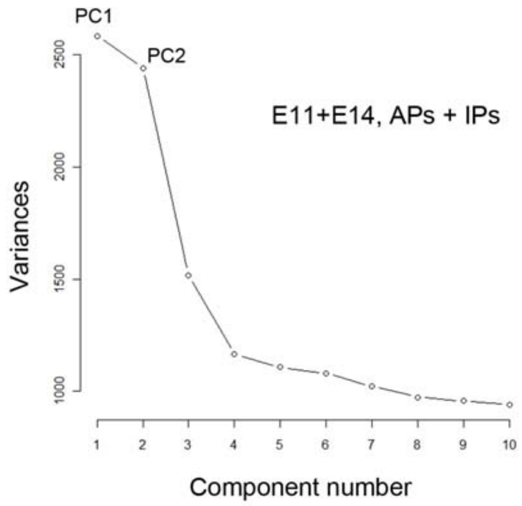

Supplementary Figure 9. Scree plot of PCA for all progenitors of E11 and E14 in Fig3a.

There is a ‘break’ between PC2 and PC3 that separates those with relatively large variances and those with small variances. At a minimum, this suggests that PC1 and PC2 are meaningful. Proportion of variances: 0.0394 for PC1, 0.0372 for PC2.

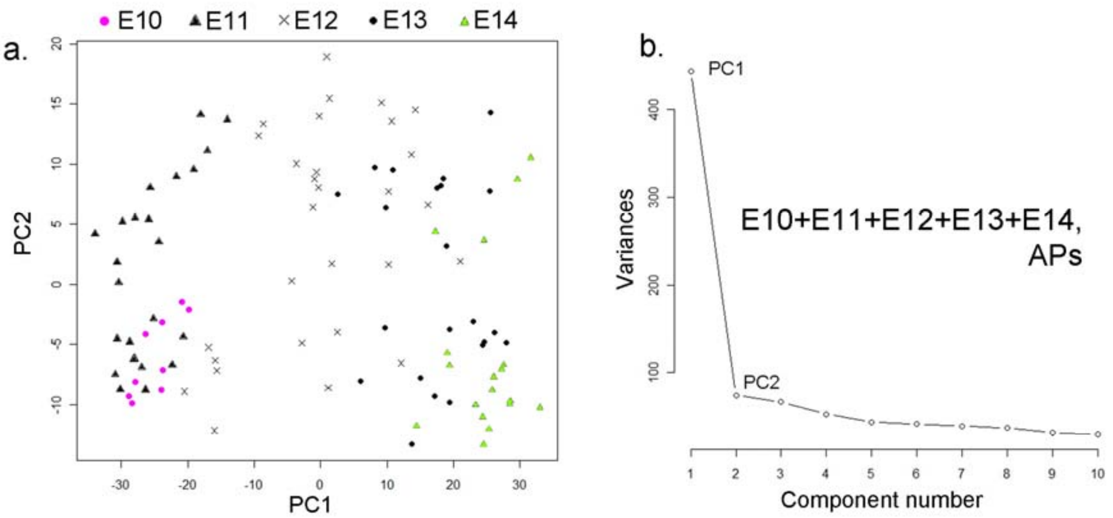

c.

Gene	PC1
Crabp2	−0.272
Fndc3c1	−0.256
Lrrn1	−0.247
Sulf2	−0.242
Flrt3	−0.208
Dmrt3	−0.204
Dmrta1	−0.188
Chst2	−0.160
Tubb3	−0.112

Gene	PC1
Pag1	0.340
Aldoc	0.294
Zbtb20	0.278
Ednrb	0.273
Sema5a	0.269
Ptn	0.244
Rlbp1	0.237
Bai3	0.202
Epha5	0.021

18 temporal-axis genes

Supplementary Figure 10. Expression levels of '18 temporal-axis genes' represent temporal change of APs. Related to Figure 4a, e.

(a-c) PCA was performed on the expression values of the 'top18 temporal-axis genes' (9 for the positive direction and 9 for the negative direction) obtained by QPCR for all APs (E10–E14, N = 102, Fig. 4a).

(b) Scree plot of (a). There is a big 'break' between PC1 and PC2 variances, validating that the PC1 largely represents the variations in the expression level of the 'top 18 temporal-axis genes' among APs during E10 and E14. Therefore, the PC1 score of each AP works as a good indicator of its temporal status in Fig. 4e. Proportion of variance: 0.409 (PC1).

(c) Contribution of the 'top 18 temporal-axis genes' to PC1.

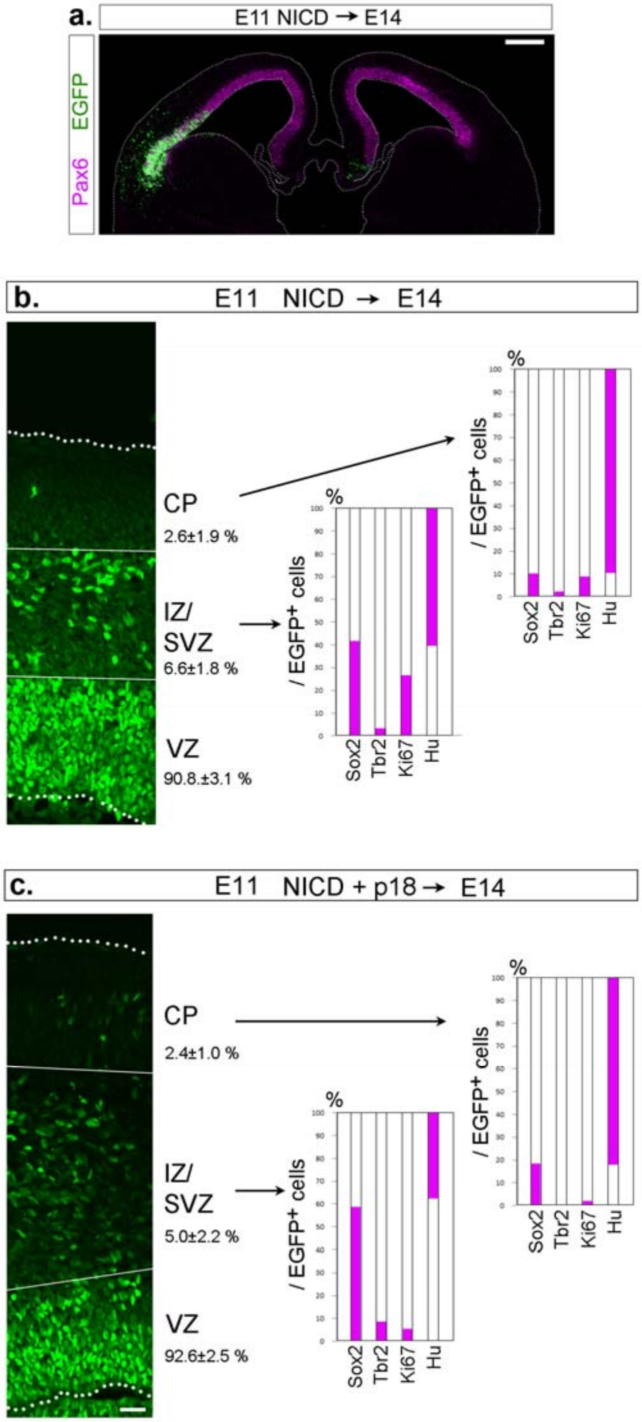

Supplementary Figure 11. Characterization of cells overexpressing NICD or NICD/p18, which migrated out of the VZ. Related to Figure 5.

(a) NICD overexpression reduced the thickness of the cerebral wall, and expanded the apical surface in the tangential direction. NICD was overexpressed with EGFP. Bar, 300μm.

(b) NICD was electroporated with EGFP at E11. After 3 days, most $EGFP^+$ cells remained in the VZ (90.8 ± 3.1%, mean ± s.d., N = 8), whereas a small population of $EGFP^+$ cells existed in the IZ/SVZ (6.6 ± 1.8%), and CP(2.6 ± 1.9%). Approximately 60% of the $EGFP^+$ cells in the IZ/SVZ and 90% of those in the CP were $Hu^+/Ki67^-$, suggesting that these

cells were neurons that lacked a sufficient level of NICD expression to inhibit their differentiation. The other 30% of $EGFP^{+}$ cells in the IZ/SVZ were $Sox2^{+}/Tbr2^{-}/Ki67^{+}$, suggesting that these cells were undifferentiated progenitor cells, in which NICD expression occurred too late to inhibit their migration from the VZ.

(c) NICD and p18 were electroporated with EGFP at E11. After 3 days, most $EGFP^{+}$ cells existed in the VZ (92.6 ± 2.5%, mean ± s.d., N = 8), whereas small population of $EGFP^{+}$ cells also existed in the IZ/SVZ (5.0 ± 2.2%), and CP (2.4 ± 1.0%). Approximately 40% of the $EGFP^{+}$ cells in the IZ/SVZ and 80% of those in the CP were $Hu^{+}/Ki67^{-}$, suggesting that these cells were neurons that lacked a sufficient level of NICD expression to inhibit their differentiation. The remaining approximately 55% of $EGFP^{+}$ cells in the IZ/SVZ and 20% in the CP were $Sox2^{+}/Tbr2^{-}/Ki67^{-}$, suggesting that these cells were cell cycle-arrested, undifferentiated progenitor cells, in which NICD expression occurred too late to inhibit their migration from the VZ. Bar, 20μm.

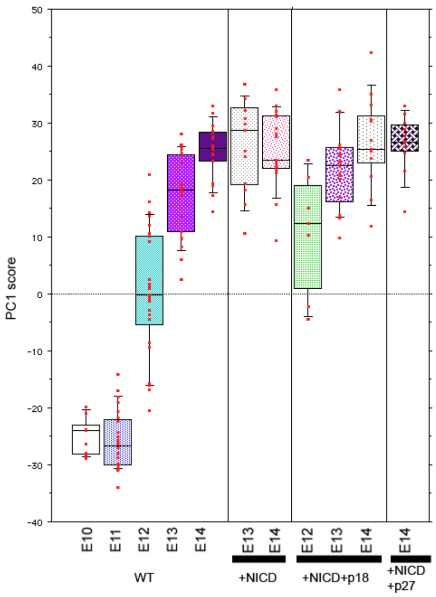

Supplementary Figure 12. Expression of the 18 temporal-axis genes in APs that co-expressed NICD and p27. NICD and p27 (Cdkn1b) were co-overexpressed at E11 by *in vivo* electroporation, and single cell cDNAs were generated at E14. Expression levels of the18 temporal-axis genes in APs (N=11) were examined by QPCR. The PC1 score of each cell was calculated as in Figure 4, and compared with those of WT cells and the cells expressing NICD/P18 (the same data in Fig. 4).

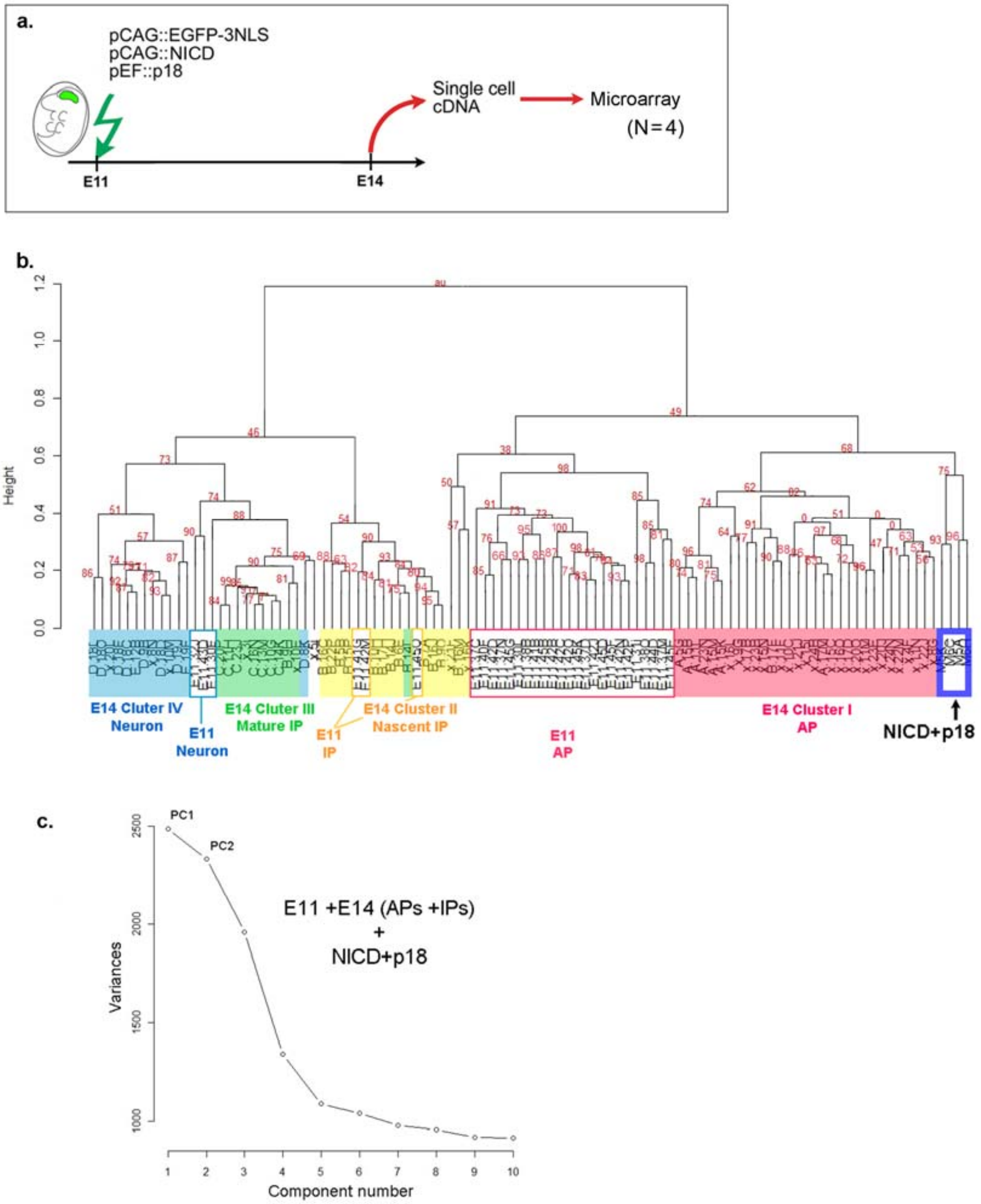

Supplementary Figure 13. Microarray analysis on single cell cDNAs of NICD/p18 co-expressing cells.

(a) Experimental design. pCAG::NICD and pEF::p18 (with pCAG::EGFP-3NLS) were electroporated into the E11 cerebral wall *in vivo*, and at E14, single cell cDNAs of $EGFP^{+}$ cells were generated and analyzed by microarrays (N = 4 cells).

(b) E14-based hierarchical clustering analysis of single cell cDNAs using the SigABC genes. The values in red at the branches are AU *p*-values (%). In this dendrogram, NICD/p18 co-expressing cells locate in the cluster (blue square) next to that of E14 APs (red).

(c) Scree plot of Fig. 4i PCA. Proportion of variances: 0.0376 for PC1, and 0.0352 for PC2.

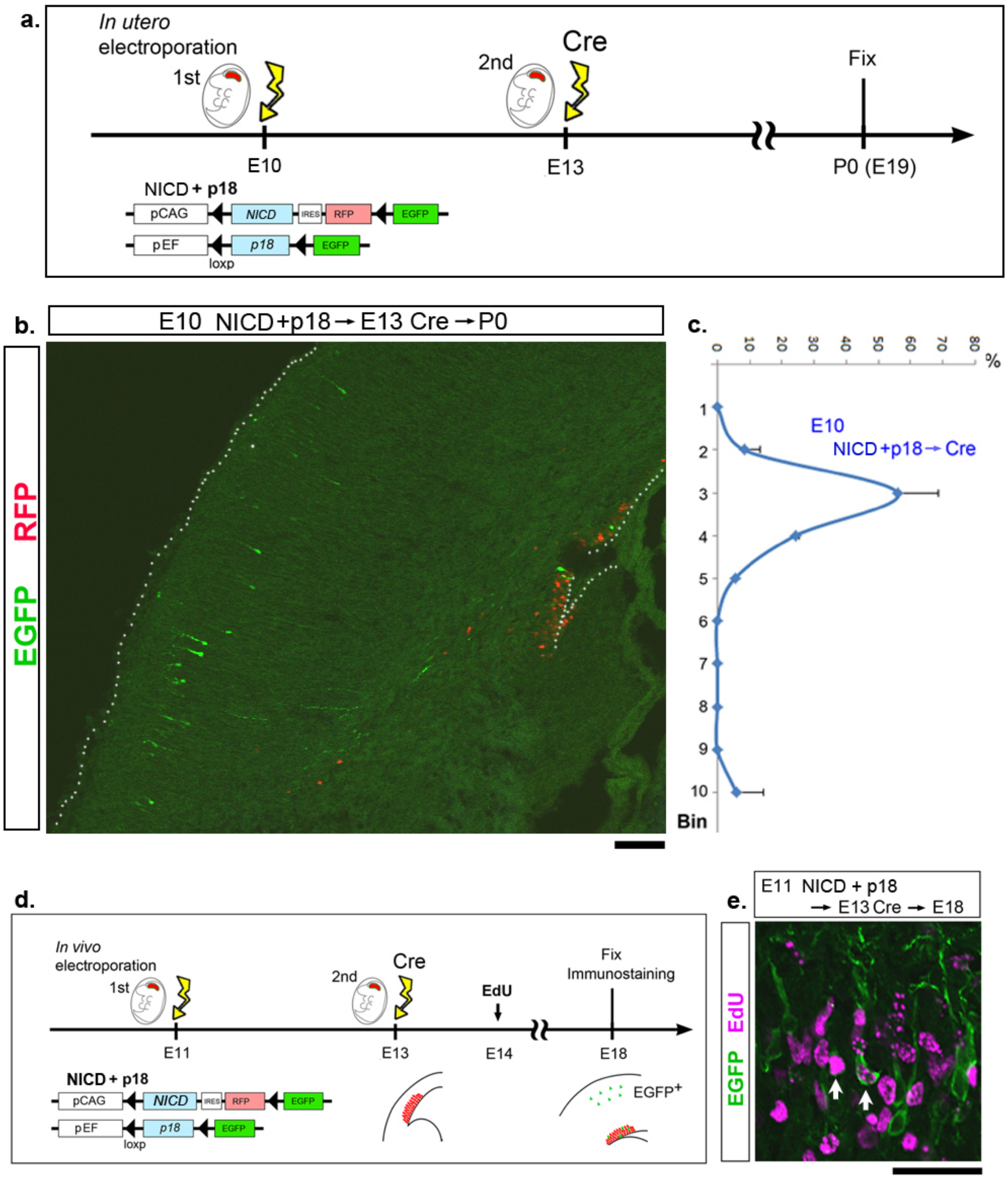

Supplementary Figure 14. Laminar fate of progenitor cells was not altered by transient cell-cycle arrest.

(a) Experimental design of double *in vivo* electroporation study. Experiments were similar to those shown in Fig. 6, but the initial electroporation was performed at E10.

(b) P0 brain section from E10 NICD/p18 and E13 Cre double-electroporated mouse. $EGFP^+$ cells that had undergone recombination to halt NICD/p18 expression were present in the CP, whereas RFP^+ (thus $NICD^+$/$p18^+$) cells were still confined to the ventricular zone. Bar, 100 µm.

(c) Distribution of $EGFP^+$ cells in the CP. CP was separated into 10 bins. Bin 1 represents the most exterior portion of the CP.

(d) Experimental design of (e).

(e) E18 brain section from E11 NICD/p18 and E13 Cre double-electroporated mouse, showing some $EGFP^+$ cells (arrows) were EdU^+ after labeling at E14. $\%EdU^+$ in $EGFP^+$ CP cells = 15.1 ± 1.9 (mean ± s.d., N = 4 embryos; > 100 $EGFP^+$ cells/embryo were examined). Bar, 30 μm.

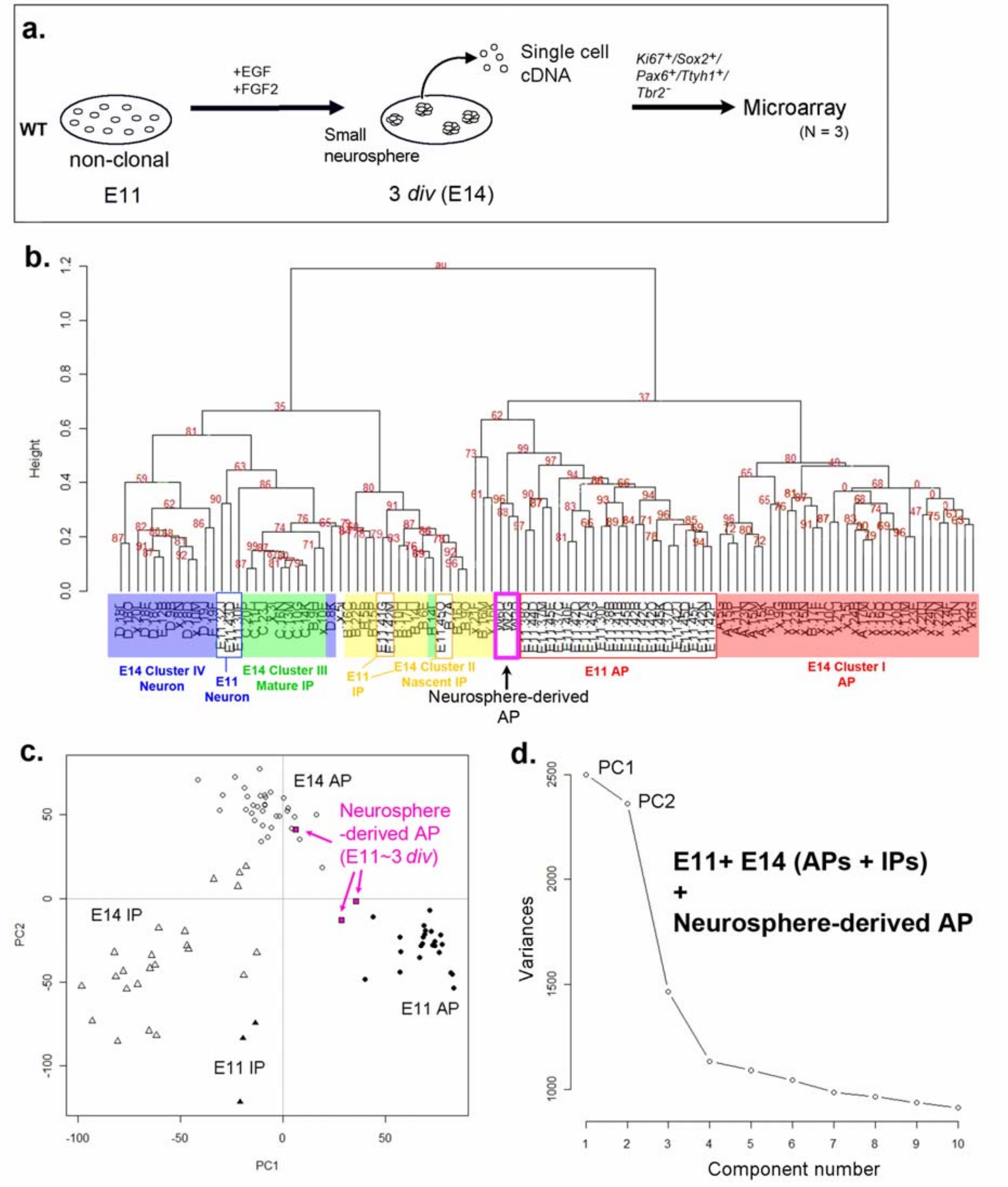

Supplementary Figure 15. Microarray analysis of single cell cDNAs from neurospheres. Related to Fig 7.

(a) Experimental design. E11 cerebral cells were cultured with EGF and FGF2 on suspension culture dishes. After 3 days, small neurospheres were formed, and single cell cDNAs were generated from the cells in the neurospheres. Then, single cell cDNAs from APs were selected by QPCR as Ki67^{+}/Sox2^{+}/Pax6^{+}/Ttyh^{+}/Tbr2^{-}, and analyzed by microarray (N = 3 cells).

(b) E14-based hierarchical clustering analysis of single cell cDNAs using the SigABC genes. The values in red at the branches are AU *p*-values (%). In this dendrogram, the neurosphere-derived APs (magenta square) locate in the AP cluster.

(c) PCA was performed using the microarray data from single-cell cDNAs from the neuroshere-derived APs (N = 3, red arrowed) and E11 and E14 progenitor cells (APs+IPs, N = 79; 17192 probe sets). Each symbol represents one cell. The plots for the neurosphere-derived APs distributed sparsely between E11 APs and E14 APs, suggesting that the temporal change in global gene expression is more various in the neurosphere-dirived APs than those *in vivo*.

(d) Scree plot of (c). Proportion of variances: 0.0383 for PC1, 0.0362 for PC2.

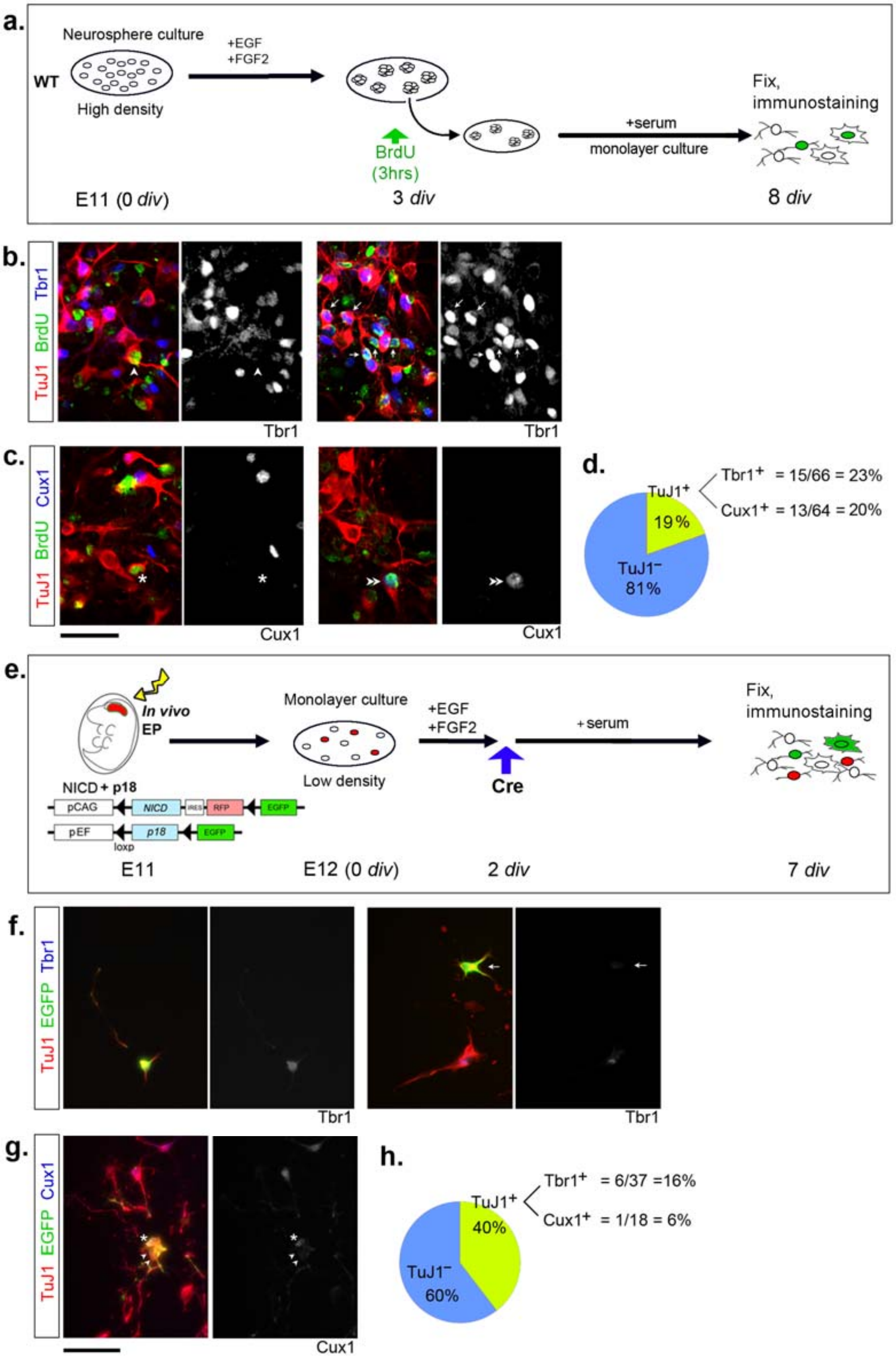

Supplementary Figure 16. *In vitro* differentiation of neurospheres and transient cell-cycle arrested cells. Related to Figure 7.

(a) Experimental design of neurosphre differentiation assay (b–d). Small neurospheres (Supplementary Figure 16) were allowed to differentiate for further 5 days with serum on PEI-coated dishes. To label the progenitor cells, BrdU was added into the medium for 3 hrs on the first differentiating day.

(b, c) Triple immunostating of the differentiation culture at 8 *div* with TuJ1 (anti-Tubb3), anti-BrdU and anti-Tbr1 (b) or anti-Cux1 (c). Examples of $Tbr1^-$ cell (arrowhead), $Tbr1^+$ cells (arrows), $Cux1^-$ cell (asterisk) and $Cux1^+$ cells (double-arrowheads) in $BrdU^+$ /$TuJ1^+$ cells were shown. Bar, 30μm.

(d) Frequency of immunoreactive cells in the $BrdU^+$ cells at 8 *div* (N = 683), suggesting that APs in neurospheres do not precisely reproduce the *in vivo* temporal progression of the AP character (Figure 6).

(e) Experimental design of *in vitro* differentiation of transiently cell-cycle arrested cells. At E11, *in vivo* electroporation was performed to express NICD/p18. After 1 day, the cerebral cells were harvested and cultured in monolayer at a low density ($3\sim6 \times 10^3$ cells/ml) with EGF and FGF2. After 2 *div*, adenovirus (Adex-CAG-NL-Cre) was infected to express Cre. Cells were fixed at 7 *div*, and examined by immunocytochemistry (f, g).

(f) $Tbr1^+$ (left) and $Tbr2^-$ (arrow) $EGFP^+$ cells.

(g) $Cux1^+$ (asterisk) and $Cux1^-$ (arrowheads) $EGFP^+$ cells. Bar, 50μm.

(h) Frequency of immunoreactive cells in $EGFP^+$ cells (N = 139). The rate of Cux1+ neuron production was quite low compared with that observed in the *in vivo* experiments (Figure 6h).

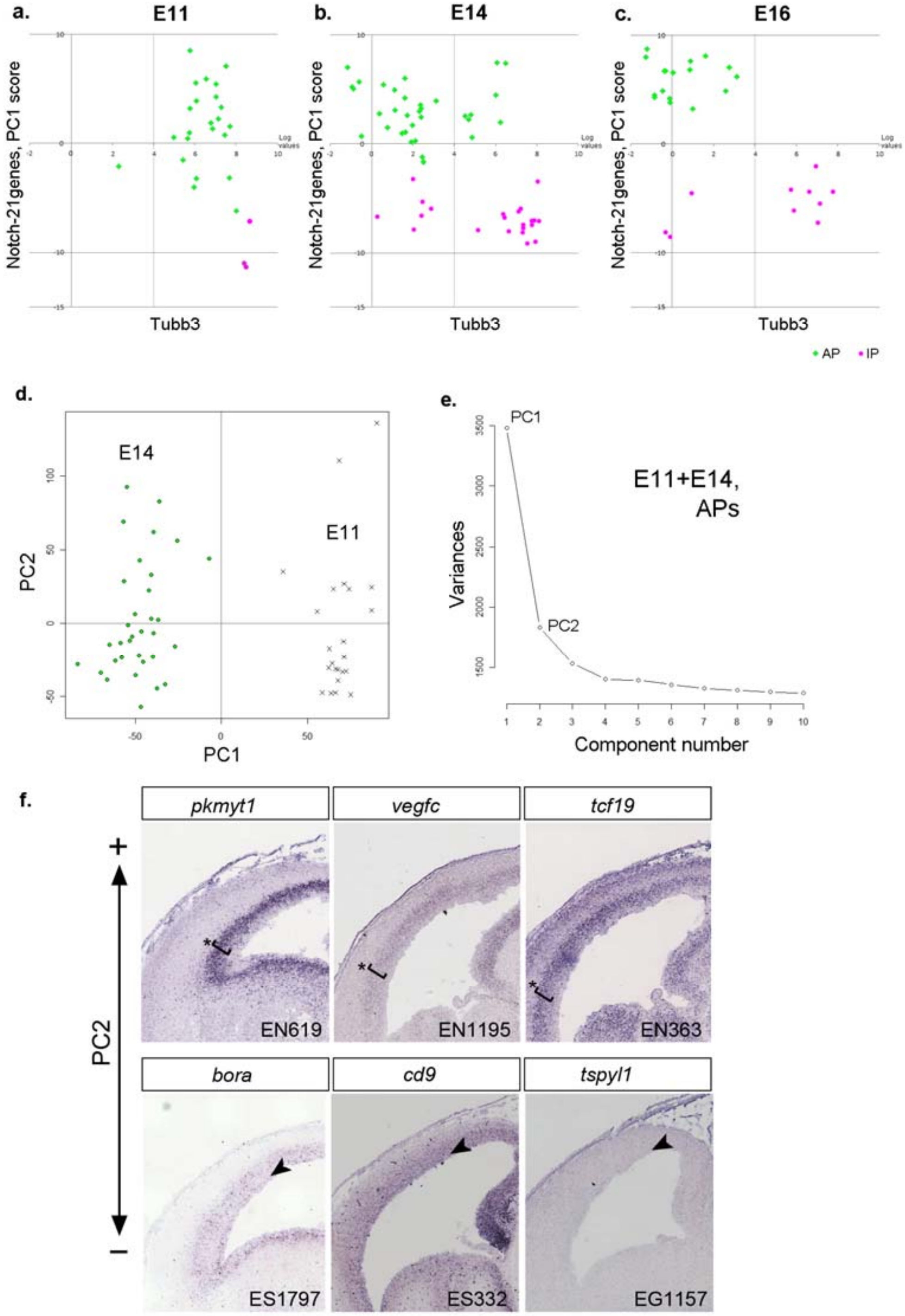

Supplementary Figure 17. Gene expression variation in APs population.

(a-c) Tubb3 expression levels do not represent 'Notch signaling status' among E11 APs. Scattered plot showing Tubb3 expression levels (probe set ID: 1415978_at) and the PC1 score of PCA on the 21-Notch signaling related genes (Supplementary Fig. 4). Each dot represents one progenitor cell. Almost all E11 APs express Tubb3 at a moderate level, although not as highly as in neurons. This is the characteristic feature of E11 APs, not of E14 and E16 APs. We did not find any particular subclasses among E11 APs, even after comparing Tubb3-high APs with Tubb3-low APs. This was also

the case for the comparison between APs showing a high Notch-PC1 score and those with a low Notch-PC1 score (not shown).

(d) The score plot of PCA on E11 and E14 APs shows heterogeneity in global gene expression both in the E11 and E14 AP populations. One symbol indicates one cell. PC1 is most likely to represent the temporal transition of APs. Proportion of variances: 0.0547 for PC1 and 0.0288 for PC2.

(e) Scree plot of (d).

(f) Expression pattern of the genes that highly contribute to PC2 (d) positively (*pkmyt1, vegfc, tcf19*) or negatively (*bora, cd9, tspyl1*) in the E14 telencephalon. PC2-positive genes peak at the basal half of the VZ (asterisk) and PC2-negative genes peak near the ventricular surface (arrowheads). This complementary expression pattern in the VZ was also seen in the genes that highly contribute to PC3 positively or negatively. These results, together with functional annotation clustering of top 40 probe sets contributing PC2 or PC3, suggested that PC2 and PC3 represent the cell cycle phase of the cells. Data from GenePaint database (http://www.genepaint.org): set ID are indicated at the bottom of the panels.

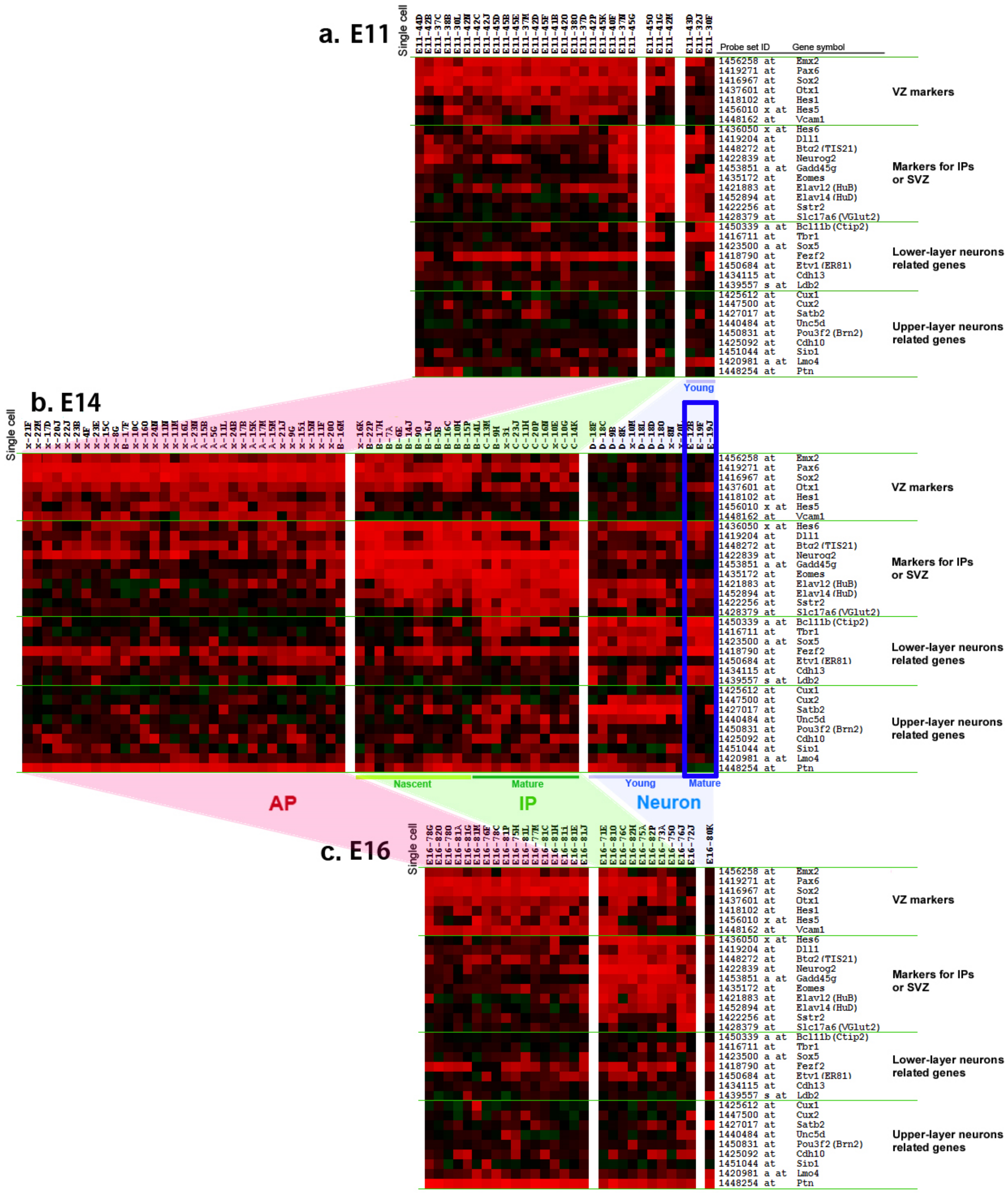

Supplementary Figure 18. Expression of layer-marker genes in single-cell cDNAs

Summary of expression levels of progenitor and neuronal layer-marker genes in single cell cDNAs at E11 (**a**), E14 (**b**) and E16 (**c**). Each column indicates one cell, and each row indicates one probe set on the microarray. The expression levels are color-coded from red (high) to green (low). At E14, all young neurons co-express both lower-layer and upper-layer marker genes, whereas mature neurons (single-cell cDNAs generated from the cortical plate fragment) specifically express lower-layer markers.

Supplementary Table 1. Marker gene expression in cDNAs from single cerebral cells, determined by QPCR.

	E11		E14		E16	
Cell type	*N*	(*N* for GeneChip)				
Hes1+ or Hes5+ progenitor cell						
Ngn2-/Tbr2-	40	(13)*	20	(18)	21	(14)
Ngn2+/Tbr2-	20	(11)	15	(12)	5	(1)
Ngn2-/Tbr2+	1		4	(4)	1	(1)
Ngn2+/Tbr2+	4	(2)	13	(13)	13	(6)
Hes1-/Hes5- progenitor cell						
Ngn2-/Tbr2-	3		1		3	(1)
Ngn2+/Tbr2-	1		1	(1)	1	
Ngn2+/Tbr2+	2	(1)	13	(9)	4	(2)
Ki67-/CyclinE1- cell (putative neuron)	4	(3)	25	(10)	14	(3)
CP neuron			9	(3)		
Other			1	(1)		
Total	75	(30)*	102	(70)	62	(28)

*This includes one sample, E11-31i, which is excluded from the analysis except for Fig1 because of its low quality.

CP, cortical plate

Supplementary Table 2.
Functional annotation clustering of top 150 'PC1 genes' of Fig. 2c

a) E11 AP > E14+E16 AP

Category	Term	P-Value	Example of PC1 genes
Annotation Cluster 1	Enrichment Score: 3.07		
GOTERM_CC_FAT	GO:0005730~nucleolus	$1.91x10^{-5}$	Tsr1, Pno1, Pop5, Dkc1, Ddx54, Wdr3, Wdr12, Cirh1a, Wdr43, Ppan, Nhp2
GOTERM_BP_FAT	GO:0022613~ribonucleoprotein complex biogenesis	$7.10\ x10^{-5}$	
GOTERM_BP_FAT	GO:0042254~ribosome biogenesis	$1.84x10^{-4}$	
GOTERM_BP_FAT	GO:0006364~rRNA processing	$2.20\ x10^{-4}$	
GOTERM_BP_FAT	GO:0016072~rRNA metabolic process	$2.35\ x10^{-4}$	
GOTERM_BP_FAT	GO:0034470~ncRNA processing	$1.15x10^{-3}$	
GOTERM_BP_FAT	GO:0034660~ncRNA metabolic process	$1.15x10^{-3}$	
GOTERM_CC_FAT	GO:0005732~small nucleolar ribonucleoprotein complex	$4.25\ x10^{-3}$	
GOTERM_BP_FAT	GO:0006396~RNA processing	$5.20\ x10^{-3}$	
Annotation Cluster2	Enrichment Score: 2.92		
GOTERM_CC_FAT	GO:0005730~nucleolus	$1.91\ x10^{-5}$	Tsr, E2f3, Dkc, Ddx54, Wdr3, Wdr43, Wdr12, Matr3, Nhp2, Pbx2, Pno1, Dach1, Pop5, Aldoa, Ccnd1, Cirh1a, Bnip3, Ppan
GOTERM_CC_FAT	GO:0031981~nuclear lumen	$7.66\ x10^{-5}$	
GOTERM_CC_FAT	GO:0043233~organelle lumen	$4.46\ x10^{-4}$	
GOTERM_CC_FAT	GO:0031974~membrane-enclosed lumen	$6.52\ x10^{-4}$	
GOTERM_CC_FAT	GO:0070013~intracellular organelle lumen	$1.26\ x10^{-3}$	
Annotation Cluster 3	Enrichment Score: 1.87		
KEGG_PATHWAY	mmu05212:Pancreatic cancer	$1.78\ x10^{-3}$	Msh6, Ccne, E2f3, Cdc42, Cblb,Ikbkb, Ccnd1, Vegfc
KEGG_PATHWAY	mmu05200:Pathways in cancer	$8.23\ x10^{-3}$	
Annotation Cluster 4	Enrichment Score: 1.34		
BIOCARTA	m_cellcyclePathway:Cyclins and Cell Cycle Regulation	$1.22\ x10^{-2}$	Rhob, Cdt1,Ccne1,Cdc42,Ccnd1, Ccnd3
KEGG_PATHWAY	mmu04110:Cell cycle	$1.37\ x10^{-2}$	
GOTERM_BP_FAT	GO:0051726~regulation of cell cycle	$2.17\ x10^{-2}$	
Annotation Cluster 5	Enrichment Score: 1.27		
GOTERM_MF_FAT	GO:0000166~nucleotide binding	$4.06\ x10^{-3}$	Galk1,Acvr2b, Mcm6, Igf2bp1 ,Stk39, Hisppd1, Gmps, Matr3, Dach1,,Cdc42, Asns, Eif5, Psmc6, Nt5c, Ryk, Brsk2, Pabpc4, Ddx54, Pak3, Msh6, Rhob, Igf2bp2,Ikbkb, Tubb3, Hsp110, Myo5b, Ror2

b) E11 AP < E14+E16 AP

Category	Term	P-Value	Example of PC1 genes
Annotation Cluster 1	Enrichment Score: 3.54		
GOTERM_MF_FAT	GO:0001871~pattern binding	$5.60\ x10^{-8}$	Ccdc80, Bcan, Ncan, Clu, Fstl1, Fstl5, Slc1a3, Thsd4, Ptn, Ptprz1, Tnc, Cyr61, Thbs1, Ptx3, Smpdl3a, Bmper, Mfge8, Lipg, LOC100047936, Vit
GOTERM_MF_FAT	GO:0030247~polysaccharide binding	$5.60x10^{-8}$	
GOTERM_MF_FAT	GO:0005539~glycosaminoglycan binding	$3.26x10^{-7}$	
GOTERM_MF_FAT	GO:0030246~carbohydrate binding	$9.60\ x10^{-5}$	
GOTERM_MF_FAT	GO:0008201~heparin binding	$1.24\ x10^{-4}$	
GOTERM_CC_FAT	GO:0005578~proteinaceous extracellular matrix	$3.57\ x10^{-4}$	
GOTERM_CC_FAT	GO:0031012~extracellular matrix	$4.64\ x10^{-4}$	
GOTERM_BP_FAT	GO:0010811~positive regulation of cell-substrate adhesion	$6.20\ x10^{-4}$	
GOTERM_CC_FAT	GO:0044421~extracellular region part	$1.83\ x10^{-3}$	
GOTERM_BP_FAT	GO:0010810~regulation of cell-substrate adhesion	$1.98\ x10^{-3}$	
GOTERM_BP_FAT	GO:0045785~positive regulation of cell adhesion	$2.43\ x10^{-3}$	

GOTERM_CC_FAT	GO:0005576~extracellular region	3.45 x10^{-3}	
Annotation Cluster 2	Enrichment Score: 2.70		
GOTERM_BP_FAT	GO:0007155~cell adhesion	7.19 x10^{-4}	Tnc, Cyr61, Vcl, Thbs1, Bcan, Nrcam, Ncan, Vcam1,Pcdh10, Pcdh8, Mfge8, Sorbs1
GOTERM_BP_FAT	GO:0022610~biological adhesion	7.30 x10^{-4}	
Annotation Cluster 3	Enrichment Score: 1.50		
GOTERM_BP_FAT	GO:0019226~transmission of nerve impulse	2.90 x10^{-3}	Shc3, Hexb, Atp1a2, Ncan, Nrcam, Cttnbp2, Pmp22
Annotation Cluster 4	Enrichment Score: 1.37		
GOTERM_BP_FAT	GO:0030030~cell projection organization	1.48x10^{-2}	Vcl, Ptprz, Wasf2, Epha4, Nrcam, Sema5a, Clu
GOTERM_BP_FAT	GO:0032989~cellular component morphogenesis	2.26 x10^{-2}	
GOTERM_BP_FAT	GO:0048812~neuron projection morphogenesis	2.45 x10^{-2}	
GOTERM_BP_FAT	GO:0048667~cell morphogenesis involved in neuron differentiation	2.72 x10^{-2}	
GOTERM_BP_FAT	GO:0048666~neuron development	3.61 x10^{-2}	
GOTERM_BP_FAT	GO:0048858~cell projection morphogenesis	3.78 x10^{-2}	
GOTERM_BP_FAT	GO:0000904~cell morphogenesis involved in differentiation	4.39 x10^{-2}	
GOTERM_BP_FAT	GO:0032990~cell part morphogenesis	4.39 x10^{-2}	
GOTERM_BP_FAT	GO:0000902~cell morphogenesis	4.43 x10^{-2}	
GOTERM_BP_FAT	GO:0031175~neuron projection development	4.77 x10^{-2}	
Annotation Cluster 5	Enrichment Score: 1.35		
GOTERM_CC_FAT	GO:0005924~cell-substrate adherens junction	5.60 x10^{-3}	Vcl, Sorbs, Cttnbp, Tns3
GOTERM_CC_FAT	GO:0030055~cell-substrate junction	6.97 x10^{-3}	
GOTERM_CC_FAT	GO:0005912~adherens junction	2.49 x10^{-2}	
GOTERM_CC_FAT	GO:0070161~anchoring junction	3.63 x10^{-2}	
GOTERM_CC_FAT	GO:0005925~focal adhesion	4.47 x10^{-2}	

Supplementary Table 3. Gene-specific primers used for QPCR of single-cell cDNA

Gene	5'-primer	3'-primer
bacillus Lys	GCCATATCGGCTCGCAAATC	AACGAATGCCGAAACCTCCTC
bacillus DAP	CCAGACCGCGGCCTAATAATG	CGCTTCTTCCACCAGTGCAG
bacillus Phe	TGAGCTCTAGGCCCAAAACGAC	TCCGGTTTTAGTCGGACGTG
bacillus Thr	GCCGATGCCGTAAAAGCAAG	CAGCTCAGGCACAAGCATCG
GAPDH	ATGAATACGGCTACAGCAACAGG	CTCTTGCTCAGTGTCCTTGCTG
ALDOA	TTCAGGCTCTTTCCCATCACTCTTGC	AGCATTCACAGACAACACCGCACACG
Beta actin	CAGCAAGCAGGAGTACGATGAGTC	CAGTAACAGTCCGCCTAGAAGCAC
PABPN1	ACCAGGCATCAGCACAACAGACCG	CCACTGTAGAATCGAGATCGGGAGCTG
Ki67	GCTTTGAGCTTTCCTGGTCATACTC	GCTTTATTGGATAGGACAGAGGGC
Ccne1	TGAGTGCTCCAGAAGCTGCTAAGG	TGTCATCTGTGTGAAGAGTCCAGTG
Pax6	ACAACACAGGCTGTTGGATCGC	GGCAAATCTTGTCGATCATGGTTTCC
HES1	TCCTAACGCAGTGTCACCTTCCAG	CCAAGTTCGTTTTTAGTGTCCGTC
HES5	TTCCTTTGTATGGGTGGGTGC	GAAGCCTTCAGAACAGCCTGTG
Neurog2	GTCAAAGAGGACTATGGCGTGTG	TACAGTCTTACGAGGTTCCCCACG
Eomes (Tbr2)	CAAAGGCATGGGGGCTTATTATGC	CAAAACACCACCAGGTCCATCTGG
Ttyh1	TTGTGTGGTAAAGCAAATGGGC	CGTAACCCAGAGCATCATGTTGC
Sox2	CATGAGAGCAAGTACTGGCAAG	CCAACGATATCAACCTGCATGG
EGFP	GACGGCGACGTAAACGGCCA	CAGCTTGCCGGTGGTGCAGA
**E11>E14 genes**		
Crabp2	GCACCAGGGTCTACGTCCGAGA	TAGCGGGCACGGAAGTCGTC
Dmrt3	TGCCAACCGACTATGAGCAGGGA	TGTCTCTGAAAACGGCCCGAGC
Dmrta1	TGTGTTACCAGGAAGGGTTCCTTAC	AAAGCTGTAAAGAAACAAGCAGGCA
Lrrn1	CCTGTGCTTGACCCCTTTCTTCTGAAG	CCAAACCCTATGTTAGCTGTTGAGAGG
Flrt3	ACAGAATAATTGTGCTTGCTTTCGGGA	TGGGGTTGTTTCGGACTCATGTGC
Tubb3	CCACCCCGTGGGCTCAAAATG	CCGAGATGCGTTTGAACAGCTCCT
Fndc3c1	TGGGGGCCATATAGTCCCAGTGC	CACTGCCTTTGCCATGCCCTGAA
Sulf2	ATGACCGCTCACACGTAACC	ATTGACACCGCGACTCTTGA
Chst2	TGGCCACTGTCAGAAAGTCC	TCCAGCCACAACTATACAGTGC
**E11<E14 genes**		
Zbtb20	TCCCAGTCCCCCTTGGATGGTG	GCTAGAGATCCAACCAGTGGACCTCC
Ednrb	ACCCAGGCCACATGTTGAAAATGAGC	ACCTCATTGTCCCGTTTGGGTTTATTG
Rlbp1	CTGCCTGCTGACTTTGGGGGTAC	TCCCATGGCAGGGCATCTCCTC
Pag1	CTGACACGGGTGTTTGTTTTCTGTC	AGCCATCACAAGTTCAACTCCACC
Ptn	GCTCTGCACAATGCTGACTG	TCTTTGACTCCGCTTGAGGC
Bai3	ACACTGAGACTTGGGAAGCC	GCACAAGAAGGGTCCTGACA
Sema5a	TTTGCCAAGAGCACAGCATTG	AACGTTGGCAGAGAGACGAT
Epha5	GTTTCAGGTAGGCCAGAGACA	TCCTGAATTCATCCTGAGGCAG
Aldoc	ACCACAGGATGGGAGGGTAG	CTGTAAGGGCTGGTGGAGAC

SUPPLEMENTARY REFERENCES

1. Kawaguchi, A.*, et al.* Single-cell gene profiling defines differential progenitor subclasses in mammalian neurogenesis. *Development* **135**, 3113-3124 (2008).
2. Kwon, G.S. & Hadjantonakis, A.K. Eomes::GFP-a tool for live imaging cells of the trophoblast, primitive streak, and telencephalon in the mouse embryo. *Genesis* **45**, 208-217 (2007).
3. Gong, S., *et al.* A gene expression atlas of the central nervous system based on bacterial artificial chromosomes. *Nature* **425**, 917-925 (2003).
4. Smart, I.H. Proliferative characteristics of the ependymal layer during the early development of the mouse neocortex: a pilot study based on recording the number, location and plane of cleavage of mitotic figures. *J Anat* **116**, 67-91 (1973).
5. Takahashi, T., Goto, T., Miyama, S., Nowakowski, R.S. & Caviness, V.S. Sequence of neuron origin and neocortical laminar fate: relation to cell cycle of origin in the developing murine cerebral wall. *J Neurosci* **19**, 10357-10371 (1999).
